# Sesn2 Serves as a Regulator between Mitochondrial Unfolded Protein Response and Mitophagy in Intervertebral Disc Degeneration

**DOI:** 10.7150/ijbs.70211

**Published:** 2023-01-01

**Authors:** Wen-Ning Xu, Chun Liu, Huo-Liang Zheng, Hai-Xia Xu, Run-Ze Yang, Sheng-Dan Jiang, Li-Xin Zhu

**Affiliations:** 1 Department of Spinal Surgery, Orthopedic Medical Center, Zhujiang Hospital, Southern Medical University, Guangzhou 510280, China.; 2 Department of Clinic of Spine Center, Xinhua Hospital, Shanghai Jiaotong University School of Medicine, Shanghai 200082, China.; 3 Department of Orthopedics, Orthopedic Research Institute, West China Hospital, Sichuan University.

**Keywords:** Intervertebral disc degeneration, Mitochondrial unfold protein response, Sesn2, Mitophagy.

## Abstract

Mitochondrial unfold protein response (UPR^mt^) can induce mitophagy to protect cell from unfold protein. However, how UPR^mt^ induces mitophagy to protect cell is not yet clear. Herein, Sesn2 was considered to be a key molecule that communicated UPR^mt^ and mitophagy in the intervertebral disc. Silencing of Sesn2 was able to reverse the protective effects of Nicotinamide riboside (NR) on nucleus pulposus (NP) cells and inhibit mitophagy induced by UPR^mt^. UPR^mt^ upregulated Sesn2 through Eif2ak4/eIF2α/Atf4, and further induced mitophagy. Sesn2 promoted the translocation of cytosolic Parkin and Sqstm1 to the defective mitochondria respectively, thereby enhancing mitophagy. The translocation of cytosolic Sqstm1 to the defective mitochondria was dependent on Parkin. The two functional domains of Sesn2 were necessary for the interaction of Sesn2 with Parkin and Sqstm1. The cytosolic interaction of Sesn2 between Parkin and Sqstm1 was independent on Pink1 (named as PINK1 in human) but the mitochondrial translocation was dependent on Pink1. Sesn2-/- mice showed a more severe degeneration and NR did not completely alleviate the intervertebral disc degeneration (IVDD) of Sesn2-/- mice. In conclusion, UPR^mt^ could attenuate IVDD by upregulation of Sesn2-induced mitophagy. This study will help to further reveal the mechanism of Sesn2 regulating mitophagy, and open up new ideas for the prevention and treatment of IVDD.

## Introduction

Intervertebral disc degeneration (IVDD) is a pathogenic process mediated by apoptotic cells 1. Apoptosis of nucleus pulposus (NP) and annulus cells plays an important role in IVDD. The apoptosis of intervertebral disc cells destroys the homeostasis and metabolism of the intervertebral disc, including the synthesis of collagen and the production of extracellular matrix, and ultimately the intervertebral disc tissue cannot maintain the biological and mechanical integrity 2-4. Apoptosis and autophagy are common in degenerative intervertebral discs. It is caused by many reasons, such as inflammatory nutrient depletion, biological and abiotic stress, and viral infection 5. Therefore, the study of the mechanism of programmed intervertebral disc cell death will be a meaningful work.

Mitochondrial unfolded protein response (UPR^mt^) refers to the response that when the mitochondrial dysfunction occurs, the mitochondrial matrix will accumulate a large number of unfolded, misfolded, and ineffective protein-induced changes in gene transcription and cellular activity 6-8. After oxidative stress-induced mitochondrial dysfunction, the mitochondrial matrix accumulates a large number of unfolded, misfolded, and ineffective proteins, leading to nuclear encoding of targeted mitochondrial chaperone proteins, such as heat shock protein family A member 9 (Hspa9, named as HSPA9 in human), heat shock protein 70 (Hspa1a, named as HSP70 in human), and proteases including caseinolytic mitochondrial matrix peptidase proteolytic subunit (Clpp, named as CLPP in human), YME1-like 1 ATPase (YME1-like 1 ATPase, Yme1l1, named as YME1L1 in human), Mitochondrial Lon protease-Like protein (Lonp1, named as LONP1 in human) and other proteins are up-regulated, which promotes the repair of mitochondrial function 9, 10, and delays aging 11.

Data from the past 5 years indicate that Atf4 is related to the integrated stress response (ISR) 12, 13. Atf4 requires phosphorylation of eukaryotic translation initiation factor 2 α subunit (eIF2α), which is catalyzed by four kinases that respond to different cellular stresses. eIF2α kinase is activated by Eif2ak4 (named as GCN2 in human) and Eif2ak3 in the amino acid consumption and endoplasmic reticulum dysfunction, which in turn causes eIF2α to be phosphorylated in the molecular structure, and the number of phosphorylated eIF2α increases 14, 15. The increase in phosphorylation of eIF2α leads to a decrease in overall protein synthesis and preferential translation of open reading frame mRNA in the 5'untranslated region, such as Atf4 mRNA 14. A number of studies have shown that different forms of mitochondrial stress induce the expression of Atf4 and genes involved in UPR^mt^, such as mitochondrial protein homeostasis and metabolic remodeling genes 16. Atf4 and Eif2ak3(named as PERK in human) have been shown to mediate the transcriptional induction of mitochondrial protein suppressor genes and the expression of genes involved in metabolic remodeling 17.

Previous studies have reported that Eif2ak3/Eif2ak4-eIF2α-Atf4 can induce Sesn2 (named as SESN2 in human) expression 18. For example, nefinavir therapy induces Sesn2 expression 19 Atf4 ectopic expression induces transcriptional upregulation of Sesn2 expression, whereas Atf3 and Chop ectopic expression does not, suggesting that Atf4 regulates Sesn2 expression 20. In some epithelial cancer cell lines, inhibitors of mitochondrial respiratory chain complex III (Myxothiazol) and I (Piericidin A) induce transcription of the P53-responsive Sesn2 gene, which plays an important role in stress response and homeostasis regulation 20, 21. UPR^mt^ and mitophagy eliminate severely damaged mitochondria and can be activated at the same time as a response to mitochondrial dysfunction. The events engineered by UPR^mt^ and mitophagy are fairly clear (transcriptional adaptation promotes restoration of mitochondrial activity and degradation of severely damaged mitochondria) 22. Yang et al have reported that mitochondrial ROS and endoplasmic reticulum stress (ERS) induced by denervation or rotenone can cause an increased expression of Sesn2 to mediate unfold protein response (UPR) in ER and mitophagy, which protect against denervated muscle atrophy 23. However, how they coordinate to restore the mitochondrial network remains to be determined.

The selective removal or degradation of damaged mitochondria by autophagy is called mitophagy. Damaged mitochondria require timely and precise excision to maintain normal mitochondrial function 24, 25. Mitophagy can play a defensive role by selectively removing damaged and dysfunctional mitochondria from cells, maintaining mitochondrial mass and thus maintaining mitochondrial physiological functions. In conclusion, mitophagy is essential for cells to clear abnormal mitochondria under stress conditions. Increased or accumulated mitochondrial damage is associated with the development and pathogenesis of many diseases 26. Mitophagy is a double-edged sword. Excessive mitophagy aggravates advanced intervertebral disc degeneration 27, 28, while moderate mitophagy delays early and mid-term intervertebral disc degeneration 29-31. Therefore, it is of great significance to study the role of UPR^mt^ and mitophagy in IVDD and its potential connection.

In summary, we propose a research hypothesis: under pathological conditions, UPR^mt^ activates mitophagy and antioxidant activity through Sesn2 to protect the survival of Nucleus Pulposus (NP) cells. The upregulation of UPR^mt^ in the intervertebral disc cells may attenuates IVDD.

## Materials and Methods

### Patients and samples

4 Pfirrmann grade Ⅰ tissue samples were obtained from congenital scoliosis patient who had undergone HR (Hemivertebra Resection). 11 Pfirrmann grade Ⅱ tissue samples were obtained from patients who had undergone PELD (Percutaneous Endoscopic Lumbar Discectomy). 11 Pfirrmann grade Ⅲ tissue samples were obtained from patients who had undergone PELD or TLIF (Transforaminal Lumbar Interbody Fusion). 8 Pfirrmann grade Ⅳ tissue samples were obtained from patients who had undergone TLIF. IVDD samples were collected from 30 patients (14 males and 16 females; age 56.9 ± 10.8 years) with degenerative disc disease. The control samples were collected from 4 patients (3 males and 1 female; age 8.5 ± 3.0 years). Above patients signed the informed consent. This study was approved by the Human Ethics Committee of Xinhua Hospital and all protocols were performed according to Declaration of Helsinki.

### Animals

**Figure 1:**
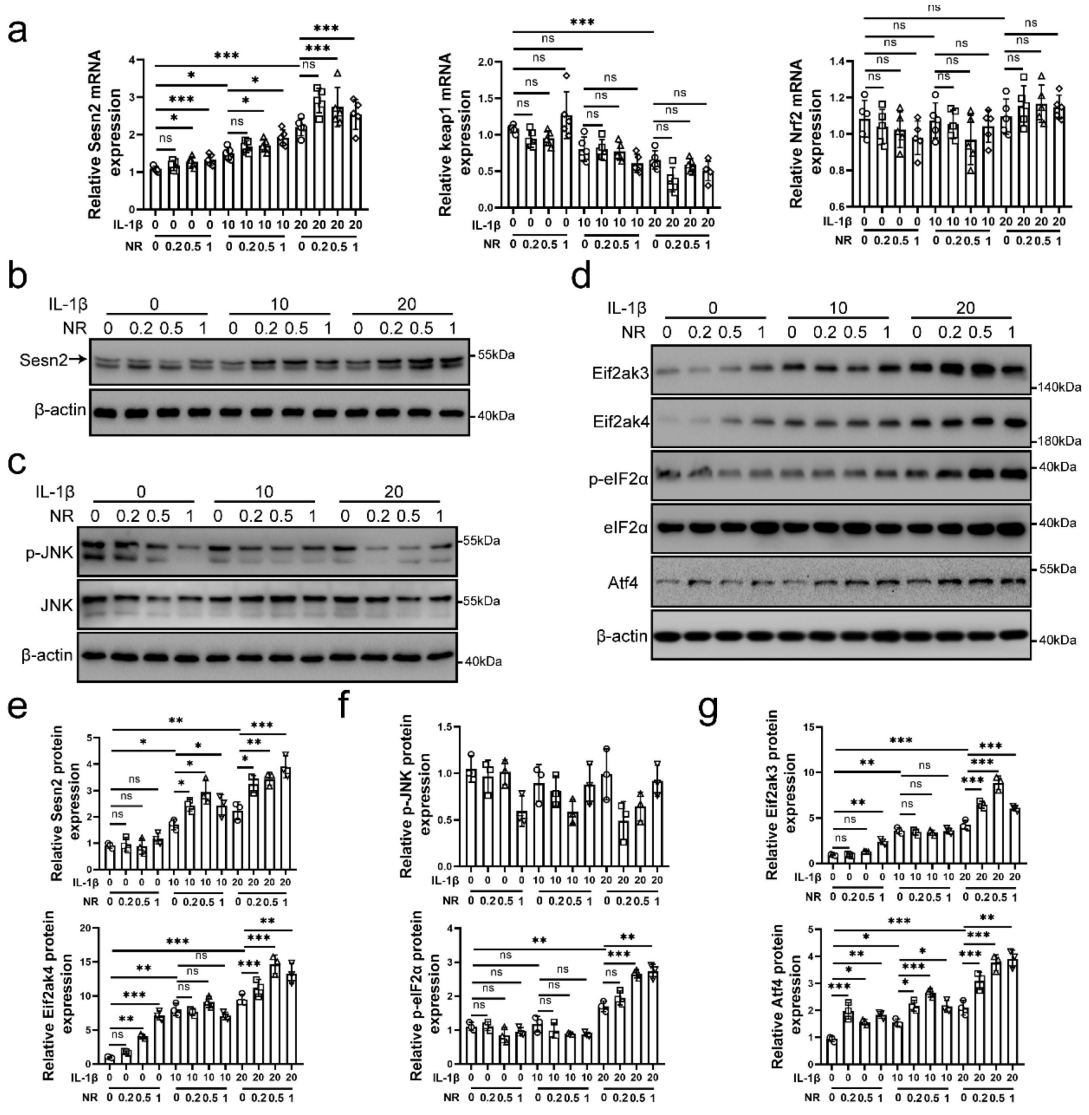
**Sesn2 and eIF2α/Atf4 signal pathway was activated by IL-1β and NR.** NP cells were divided into 3 groups and treated with 0 ng/ml, 10 ng/ml and 20 ng/ml IL-1β respectively. Each group was subdivided into 4 groups and treated with 0, 0.2, 0.5- and 1-mM NR respectively after IL-1β treatments. The experimental grouping settings were indicated. **(a)** The mRNA expression of Sesn2, Keap1 and Nrf2 in NP cells were detected by qRT-PCR (n = 5). **(b)** Sesn2 and β-actin protein expression in NP cells were detected by Western blot (n = 3). **(c)** p-JNK, JNK and β-actin in NP cells were detected by Western blot (n = 3). **(d)** Eif2ak3, Eif2ak4, p-eIF2α, eIF2α, Atf4 and β-actin in NP cells (n = 3). **(e)** The quantitative analysis of Sesn2 protein expression were showed. **(f)** The quantitative analysis of p-JNK protein expression were showed. **(g)** The quantitative analysis of Eif2ak3, Eif2ak4, p-eIF2α and Atf4 protein expression were showed. Statistical significance was analyzed by one-way ANOVA followed by a post hoc Tukey's test. All data were presented as mean ± SEM (error bars). *p < 0.05; **p < 0.01; ***p<0.001.

Sesn2 knockout (Sesn2-/-) mice was generated by CRISPR/Cas9 system (Fig 1). To obtain Sesn2-/- mice, single-guide RNAs (sgRNAs) were designed targeting exon3-exon8 by using MEGAshortscript^TM^ Kit (Thermo Fisher Scientific, Waltham, MA, USA). The sequence of sgRNAs were listed in Table 1. In order to microinject Cas9 protein and sgRNA into fertilized eggs, male C57BL6/J mice were individually caged for 1 week before mating. Female C57BL6/J mice were given 5 IU of serum gonadotropin and 5 IU of human chorionic gonadotropin (HCG) to pregnant horses for superovulation. After C57BL6/J male and female mice were injected with HCG, the fertilized eggs of male and female mice were taken for mating. sgRNA (40ng/μl) and Cas9 protein (40ng/μl) were injected into the cytoplasm of the fertilized egg at the single-cell stage using continuous flow injection microinjection. The surviving two-cell embryo was implanted into the fallopian tube of a pseudo-pregnant female. The creation mice (F0) were analyzed in the born pups. F0 mice were mated with C57BL6/J wild-type mice, and F1 mice were bred to obtain F2 homozygous mutants. In order to maintain a stable inbred background, female mice will be backcrossed to their parental inbred strains every 10 generations.

### Cell culture

NP cells were separated from Sprague-Dawley rat disc tissues. The separation methods were performed according to our previous study 32. Rat tissues were gained from male rats (6 weeks old and 200-250 g in weight). Disc was sliced as fragments. Fragments of rat dis tissues were digested with 0.1% collagenase for 4h. And then the tissues were removed as explants and placed in complete medium (DMEM/F12 and 10% fetal bovine serum (FBS) supplemented with antibiotics) at 37 °C in a 5% CO2 environment. Tissues were removed form plates after 1 week. Finally, primary passage cells were harvested by 0.25% trypsin-EDTA (1 mM) solution and replanted in appropriate culture plates once confluent.

### Plasmids

The Flag-Sestrin2, Flag-Sestrin2-NTD (N-terminal domain, 1-220), Flag-Sestrin2-linker-CTD+L (C-terminal domain + linker, 220-480) and HA-Sestrin2-linker-CTD+L (C-terminal domain + linker, 220-480) mutants were constructed by cloning the cDNA of PGMLV-CMV-MCS-PGK-Puro.The Myc-Sqstm1 and Myc-Parkin were constructed by cloning the cDNA of PGMLV-CMV-MCS-PGK-Puro. Small interfering RNAs to Atf4, Sesn2 and Pink1 (si-Atf4, si-Sesn2 and si-Parkin) were designed and purchased from Genomeditech (Shanghai, China). Si-Pink1 and Si-Sqstm1 were purchased from GenePharma (Shanghai, China). The sequences of si-Atf4, si-Sesn2 and si-Parkin were listed in Table 2. Si-Pink1 and Si-Sqstm1 were listed in Table 3. Briefly, cDNA, si-RNAs and Lipofectamine 3000 (Thermo Fisher, USA) were added to Opti-MEM medium (Thermo Fisher, USA) respectively. And then they were mixed as mixtures and subsequently incubated for 20 min. Finally, the mixtures were added to plates.

### Lentivirus transfection

NP cells were grown until 80% confluence in six-well plates. After polybrene was added to the medium, 1 μM mRFP-GFP-LC-3 and Sesn2 lentivirus were added into plates and incubated for 48 h. NP cells transfected with mRFP-GFP-LC-3 lentivirus were used to determine the flux of autophagy in follow-up experiments. The green dot represents the initial autophagosome, and the red dot represents the mature autophagosome. NP cells transfected with Sesn2 lentivirus were used to overexpress the expression of Sesn2.

### Quantitative Real-time PCR

Total RNA was obtained from NP cells and disc tissues by TRIzol reagent (TaKaRa, Inc., Dalian, China). Reverse transcription was performed to gain the first strand cDNA by using the PrimeScript RT Master Mix cDNA Synthesis Kit (TaKaRa, Inc., Dalian, China). The real-time PCR was operated on ABI 7500 system Applied Biosystems, Foster City, CA, USA) using SYBR1 Premix (TaKaRa, Inc., Dalian, China) according to manufacturer's protocol. The relative expression of target genes was quantified the 2^-ΔΔCt^ methods after normalization to GAPDH. The sequence of specific primers was listed as Table 4.

### Mitochondrial protein extraction

Mitochondrial extraction was performed by Cell Mitochondrial Isolation Kit (Beyotime, Cat. No. C3601, China). NP cells were washed with PBS, digested with Trypsin-EDTA solution, centrifugated at room temperature for 5-10 minutes. After cells were collected, Cells were added with 1-2.5ml of mitochondrial separation reagent and place in an ice bath for 10-15 minutes. And then the suspension was transferred to an appropriately sized glass homogenizer for about 10-30 homogenates. The cell homogenate was centrifuged at 600g at 4℃ for 10 min. The supernatant was transferred carefully to another centrifuge tube and centrifuged at 11,000g for 4 min. The supernatant was removed carefully and the precipitate was the isolated mitochondria. The separated mitochondrial samples were lysed by RIPA containing PMSF and were used for Western blot.

### Co-Immunoprecipitation

NP cells were added with appropriate amount of cell lysis buffer (containing protease inhibitor), lysed on ice for 30 minutes, and centrifuged at 4°C at maximum speed for 30 minutes and then collected the supernatant. A small amount of lysate was used to perform Western blot analysis and the remaining lysate were added 1μg of the corresponding antibody, and then incubated with slow shaking at 4°C overnight. 10μl of protein A agarose beads were washed 3 times with an appropriate amount of lysis buffer, and centrifuged at 3,000 rpm for 3 minutes each time. The cell lysate that had been incubated with the antibody overnight were added with the pretreated 10μl protein A agarose beads and incubated with slow shaking at 4°C for 2-4 h to couple the antibody to the protein A agarose beads. After the immunoprecipitation reaction, the lysate was centrifuged at 3,000 rpm at 4°C for 3 minutes. The supernatant was carefully aspirated and the agarose beads were washed with 1ml of lysis buffer 3-4 times. Finally, 15µl of 2×SDS loading buffer was added and boiled for 5 minutes. The samples were used to Western blot analysis.

### Western blot analysis

30 µg protein was separated by SDS-polyacrylamide gel electrophoresis and electroblotted onto polyvinylidene difluoride membranes. Membranes containing target proteins were blocked using 5% non-fat milk for 2 hours and incubated with primary antibodies overnight at 4 ℃. The information primary antibodies were listed in Table 5. Membranes were washed with TBST three times and then incubated with corresponding horseradish peroxidase-conjugated secondary antibodies at room temperature for 1 hours. The membranes of target protein were developed with ECL plus reagent (Millipore) on the ChemiDocTM XRS + System (Bio- Rad, USA).

### Flow cytometry assays

NP cell apoptosis was detected by an Annexin V-FITC apoptosis detection kit (BD, Cat. No. 556547, USA) according to manufacturer's protocol. After various treatments, NP cells were obtained and then washed by ice TBS for twice. Subsequently, 300 μl of 1 × binding buffer were added to resuspend the cells. Next, 5 μl of Annexin V-FITC and PI were added. The cells were vortexed gently, incubated for 30 min in the dark and then analyzed by flow cytometry.

### TUNEL assays for apoptosis

NP cells were seeded on coverslips in six-well plates. After various treatments, cells were washed by PBS. Cells were fixed with 4% paraformaldehyde for 30 minutes and then washed once with PBS. Cells were permeabilized by PBS containing 0.3% Triton X-100 at room temperature for 5 minutes. Cells were washed by PBS for twice. Adding 50 μL of TUNEL detection solution to the samples and incubating at 37 °C for 60 minutes in the dark were performed. And then samples were added with DAPI and incubated for 5 minutes. Finally, images were taken by fluorescence microscopy (Olympus BX51).

### ROS assay

Reactive oxygen species detection kit (Beyotime, China) was used to detect ROS production according to the manufacturer's protocol. After various treatments, NP cells were washed with PBS, incubated with DCFH-DA-containing serum-free medium for 20 min at 37°C, and then DCFH-DA was removed and washed 3 times with serum-free medium. The DCF fluorescence distribution of cells was detected by fluorescence microscopy analysis (Olympus Fluoview, Japan). Positive cells glow green.

### JC-1 assay for mitochondrial membrane potential

The mitochondrial membrane potential (mtDY) was detected by JC-1 kit (C2006; Beyotime, China) according to the manufacturer's instructions. The NP cells were obtained and re-suspended in 500ul JC-1 staining fluid, and then incubated in the dark at 37 ℃ for 20 min. Cells were re-suspended in 500ul ice-cold staining buffer and detected by fluorescence microplate after washing with ice-cold staining buffer twice and centrifugation. In normal cells, mitochondria have a high mtDY and JC-1 formed orange-red fluorescent J-aggregates, while in cells with depolarized or damaged mitochondria, the sensor dye appeared as green fluorescent monomers.

### Surgical procedure

Disc degenerative model were performed in Sesn2-/- mice as previously described 27. All the experiments were abided by International Guiding Principles for Biomedical Research Involving Animals and approved by Ethics Committee of Xinhua Hospital Affiliated with Shanghai Jiao Tong University School of Medicine. Eighteen 8-week healthy adult mice were randomly divided into three groups: Sesn2 WT group + control Lentivirus, Sesn2-/- + control Lentivirus and Sesn2-/- + Lenti-Sesn2. The disc tissues of mice were obtained in 4 months old. Twenty-four 8-week healthy adult WT and Sesn2-/- mice were randomly divided into four groups: the sham operation group (skin incision), the sham operation + NR (skin incision + NR), the disc degenerative (surgical procedure) group and the disc degenerative + NR (surgical procedure + NR) group. Mice were fed with pellets containing vehicle or NR (400 mg/kg/day) for 2 months according to previous study 33. The pellets were prepared by mixing powdered chow diet 34 with water or with NR dissolved in water. Pellets were dried under a laminar flow hood for 48 hours. The surgical procedure was performed according to previous study 27. In brief, animals were anesthetized intraperitoneally using 0.1ml 1% pentobarbital (50 mg/kg). After 5 min, the spinous processes, sacrospinal muscles, interspinous ligaments, supraspinous ligaments, and posterolateral 1/2 of bilateral zygapophysial joints of the lumbar spine were removed through a dorsal medial approach. Skin incision served as a control. 2 months after surgery, the disc tissues of rats and mice were obtained in 4 months old.

### Lentivirus-Sesn2 transfection *in vivo*

Lentivirus Sesn2 transfection was performed according to our previous study 27. Surgical procedure after one weeks, the L4-5 discs were confirmed by a trial radiograph. Needles were used to puncture the L4-5 discs through the annulus fibrosus. The needles were fixed into the nucleus pulposus and 2 ul Lentivirus was then injected into the nucleus pulposus each two weeks until the rats were sacrificed. Finally, the needles were pulled out gently and slowly.

### Safranin O-fast green staining

The disc specimens were decalcified with EDTA and fixed in 4% formaldehyde. The disc specimens were dehydrated in gradient solutions of ethyl alcohol and then embedded in paraffin. The specimens were cut into 5-μm sections continuously and then were stained with safranin O-fast green (S-O). The morphology of disc was evaluated by a blinded and experienced histological researcher.

## Statistical analysis

The data are presented as the mean ± SD (standard deviation). Statistical analyses were performed on GraphPad prism 8. Multiple comparisons of data among the groups were analyzed by one-way ANOVA and Tukey's post hoc test. Independent-samples t-tests were used to analyze the differences between two groups. Differences were considered statistically significant when the P value was <0.05. ***p < 0.001, **p < 0.01, *p < 0.05. All experiments were performed at least three.

## Results

### Sesn2 might act as a bridge between mitochondrial unfolded protein response and mitophagy

Interleukin (IL)-1β is thought to lead to the senescence and apoptosis of NP cells, resulting in IVDD 35, 36. As a NAD^+^-boosting compound, Nicotinamide riboside (NR) was reported to improving the level of UPR^mt^, which alleviates aging in mice 11, 37, 38. Therefore, we performed 0.2, 0.5 and 1mM NR to treat NP cells for 6h after IL-1β treatments (0, 10 and 20 ng/ml) 39. Sestrin 2 (Sesn2) has been reported to regulate the mitophagy 40-43. Sesn2 senses oxidative stress from mitochondria, attenuates senescence and improves the life span in mammals 44, 45. To investigate whether Sesn2 is involved in UPR^mt^, we detected the expression of Sesn2 in NP cells. As shown in Fig. 1a, b and e, IL-1β increased Sesn2 mRNA and protein expression. NR further enhanced Sesn2 mRNA and protein expression. These data showed that Sesn2 might be involved in UPR^mt^. However, the correlation between UPR^mt^ and Sesn2 are still not clear.

In recent years, some studies have reported that Sesn2 promotes p62-dependent autophagy to degrade Keap1, thereby upregulating Nrf2 signaling and reducing ROS accumulation 46-48. However, the results in this study showed that Keap1 and Nrf2 mRNA expression was not changed after NR treatments (Fig. 1a). These results confirmed that UPR^mt^ did not induce mitophagy by Sesn2/keap1/Nrf2 signal pathway. JNK signal has been reported to regulate Sesn2 directly and UPR^mt^ also regulate JNK 49, 50. Western blot results disavowal above conjecture (Fig. 1c and f). JNK was down-regulated after IL-1β treatments and NR treatments. Eif2ak3/Eif2ak4-induced eIF2α-Atf4 signal pathway was regulated by UPR^mt^. UPR^mt^ promotes the UPR^mt^ markers including Clpp, Lonp1, Hspd1 (named as HSP60 in human) and so on via Eif2ak4-eIF2α-Atf4 signal pathway 10, 16, 17. Our results confirmed this point that IL-1β increased Eif2ak3, Eif2ak4, p-eIF2α and Atf4 protein expression in NP cells and UPR^mt^ inducer NR further enhanced their expression (Fig. 1d and g). Above results demonstrated that UPR^mt^, Eif2ak4-eIF2α-Atf4 signal pathway and Sesn2 might have some correlation.

### SESN2 expression was decreased in human tissues of IVDD

**Figure 2:**
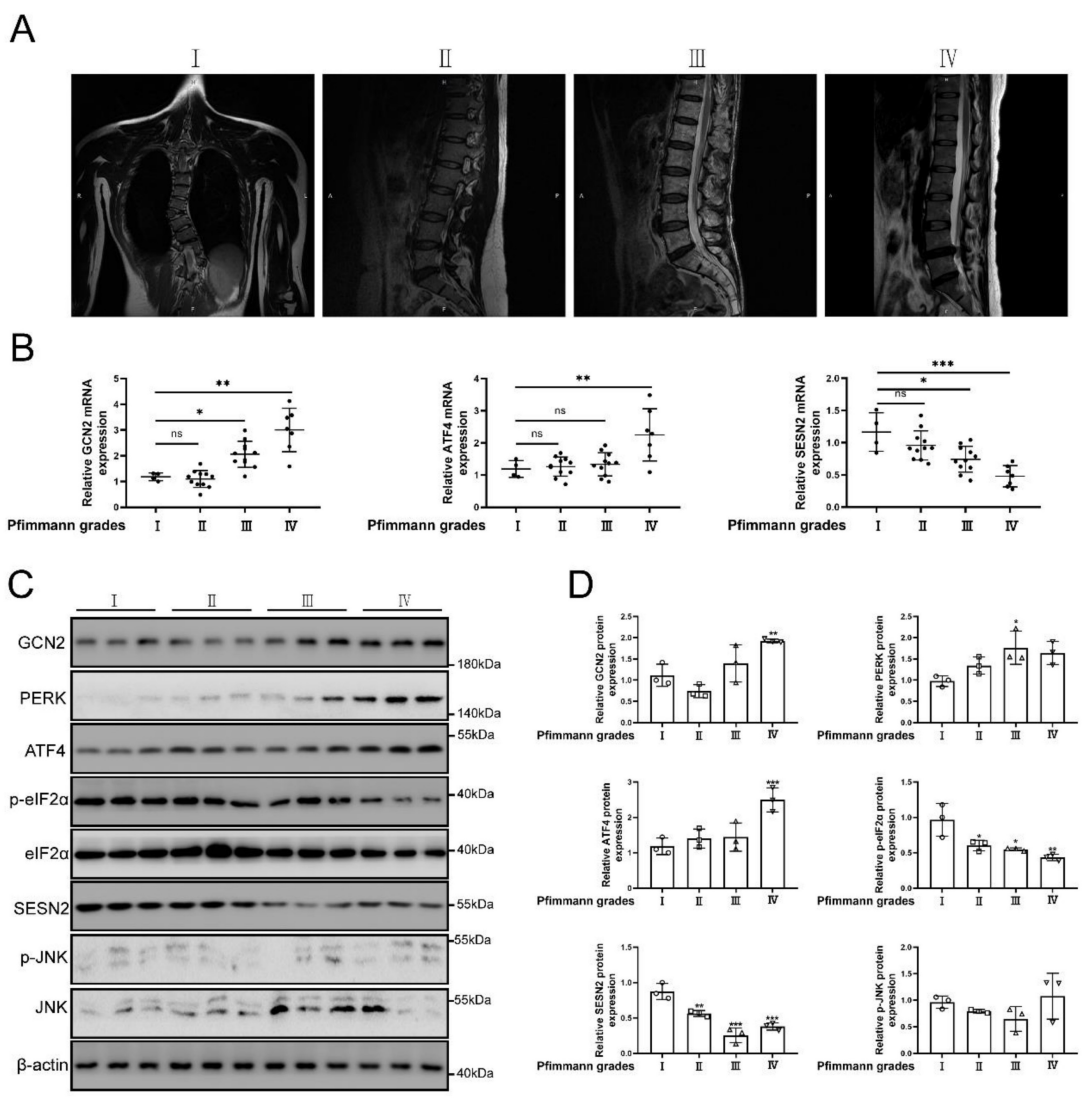
** The protein expression GCN2, ATF4 and SESN2 were confirmed. (a)** The representing graphics of each Pfirrman grade was shown. **(b)** The mRNA expression of GCN2, ATF4 and SESN2 in the tissue of human IVDD were detected by qRT-PCR (n = 5). **(c)** The protein expression of GCN2, PERK, ATF4, p-eIF2α, eIF2α, SESN2, p-JNK, JNK and β-actin in the tissue of human IVDD were confirmed by Western blot (n = 3). **(d)** The quantitative analysis of GCN2, PERK, ATF4, p-eIF2α, SESN2 and p-JNK protein expression were showed. Statistical significance was analyzed by one-way ANOVA followed by a post hoc Tukey's test. All data were presented as mean ± SEM (error bars). *p < 0.05; **p < 0.01; ***p<0.001.

Grades of MRI imaging for IVDD referenced to the Pfirrmann scoring system 51. The source of normal and degenerative intervertebral disc tissues in human was according to previous study 52. The classification of intervertebral disc tissue according to the grades of Pfirrmann scoring system. The representative MRI of patients were shown in Fig. 2a. As the degree of degeneration increased, SESN2 and p-eIF2α (named as Sesn2 in rat and mouse) expression was decreased in human tissues of degenerative intervertebral discs (Fig. 2b, c and d). The expression of GCN2, PERK and ATF4 (named as Atf4 in rat and mouse) increased in human tissues of discs. The expression of JNK did not change significantly in human tissues of discs (Fig. 2b, c and d). This was an interesting finding. Eif2ak3/Eif2ak4-eIF2α-Atf4 signal pathway was also reported to regulate Sesn2 signal pathway 18, 20, 21, 53. Based on these studies, the hypothesis that UPR^mt^ activated Sesn2 through Eif2ak3/Eif2ak4-eIF2α-Atf4 signal pathway needed to be verified according to follow-up experiments.

### Silencing of Sesn2 reduced the protective effect of Nicotinamide riboside on NP cells

**Figure 3:**
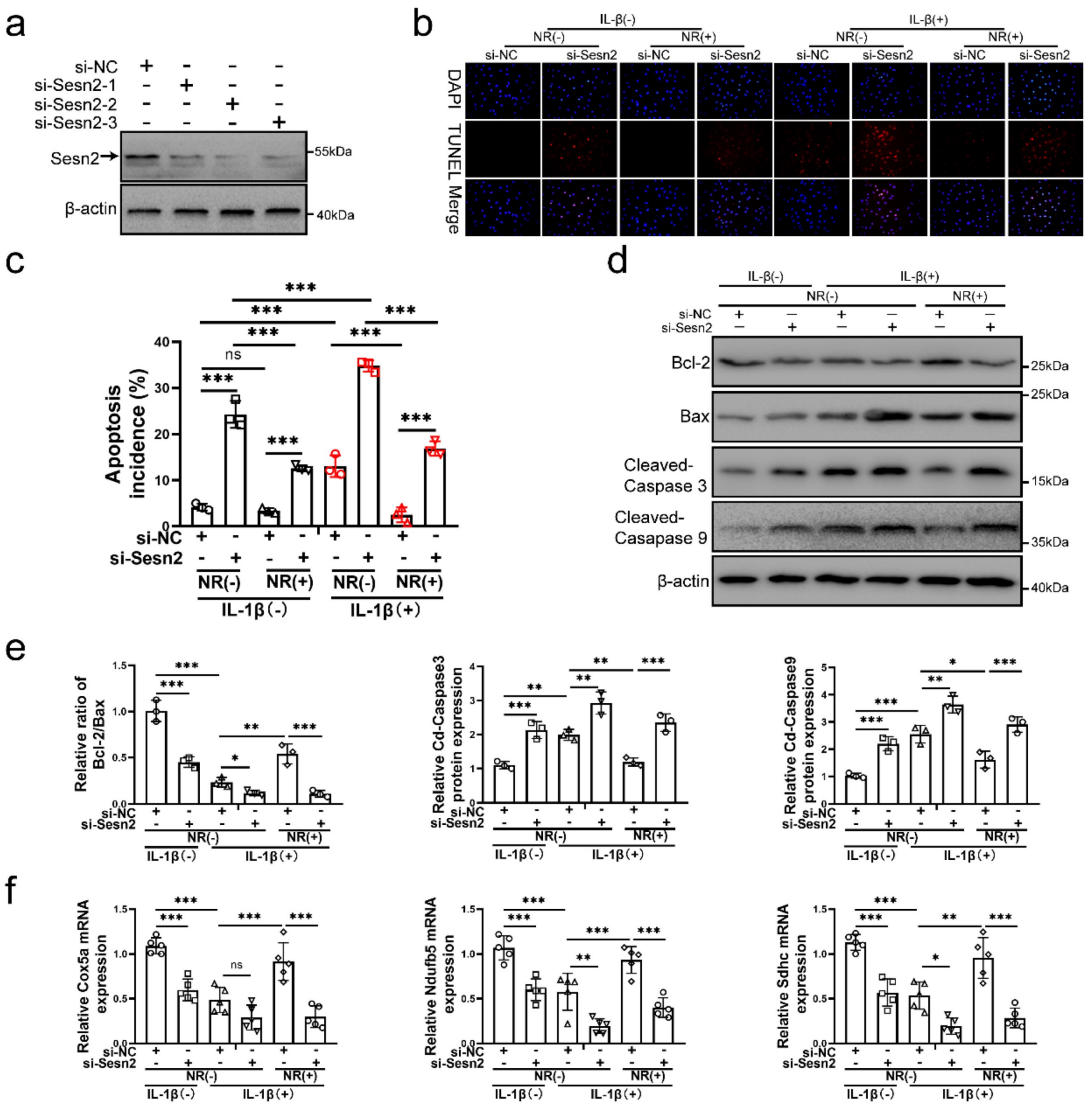
** Sesn2 knockdown reduced the protective effect of NR in NP cells**. **(a)** Western blot to Sesn2 and β-actin in NP cells (n = 3). NP cells were transfected with si-NC, si-Sesn2-1, si-Sesn2-2 and si-Sesn2-3 respectively. (b-f) NP cells were transfected with si-NC or si-Sesn2, treated with 0 ng/ml and 20 ng/ml IL-1β for 48h and then treated with 0- and 1-mM NR for 6h. The experimental grouping settings were indicated. **(b)** TUNEL assays to NP cell apoptosis (n = 3). **(c)** The bar-chart of flow cytometry to NP cell apoptosis (n = 3). **(d)** Western blot to Bcl-2, Bax, Cleaved-caspase 3, Cleaved-caspase 9 and β-actin in NP cells (n = 3). **(e)** The quantitative analysis of Bcl-2/Bax, Cleaved-Caspase 3 and Cleaved-Caspase 9 protein expression were showed. **(f)** Mitochondrial functional mNRA expression including Cox5a, Ndufb5 and Sdhc were detected by qRT-PCR (n = 5). Statistical significance was analyzed by one-way ANOVA followed by a post hoc Tukey's test. All data were presented as mean ± SEM (error bars). *p < 0.05; **p < 0.01; ***p<0.001.

To confirm whether UPR^mt^ protects NP cells via upregulating Sesn2, Sesn2 was silenced in NP cells before IL-1β and NR treatment. Sesn2 was silenced by si-Sesn2-1, si-Sesn2-2 and si-Sesn2-3 in NP cells (Fig. 3a). After Sesn2 was silenced, NR could not protect NP cells from IL-1β (Fig. 3b and c). Reduction of apoptosis by NR were partially destroyed in NP cells after Sesn2 knockdown. Regardless of whether it was treated by IL-1β, knocking down the expression of Sesn2 significantly increased the TUNEL positive rate of NP cells (Fig. 3b). Flow cytometry showed that Sesn2 knockdown increased the apoptosis rate (late + early) from 4.19 ± 0.37% to 24.30 ± 1.93% in the IL-1β (-) + NR (-) groups. IL-1β increased the rate of apoptosis (late + early) from 4.19 ± 0.37% to 13.08 ± 1.96%. NR reduced apoptosis (late + early) induced by IL-1β from 13.08 ± 1.96% to 2.51 ± 0.50%. However, Sesn2 knockdown increased the apoptosis rate (late + early) from 2.51 ± 0.50% to 16.88 ± 2.83% (Fig. 3c and Fig. S1a). The protein expression of Bcl-2, Bax and Cleaved-caspase3, Cleaved-caspase 9 bore out these results (Fig. 3d and e). To confirm the role of Sesn2 in mitochondria, some methods were used to determine mitochondrial function. Sesn2 knockdown could prevent repair of mitochondrial function induced by NR (Fig. 3f). Cox5a, Ndufb5 and Sdhc protein expression represents changes in mitochondrial function. The promotion of Cox5a, Ndufb5 and Sdhc protein expression caused by NR was reduced in NP cells after Sesn2 knockdown. The results of ROS Assay showed that Sesn2 knockdown significantly increased ROS production induced by IL-1β (Fig. S2a). JC-1 results also confirmed that Sesn2 knockdown could further increase the damage of mitochondrial membrane potential (Fig. S2b). These results showed Sesn2 served as an important role in the protection of UPR^mt^ on NP cells.

### Silencing of Sesn2 prevented mitophagy induced by mitochondrial unfolded protein response

**Figure 4:**
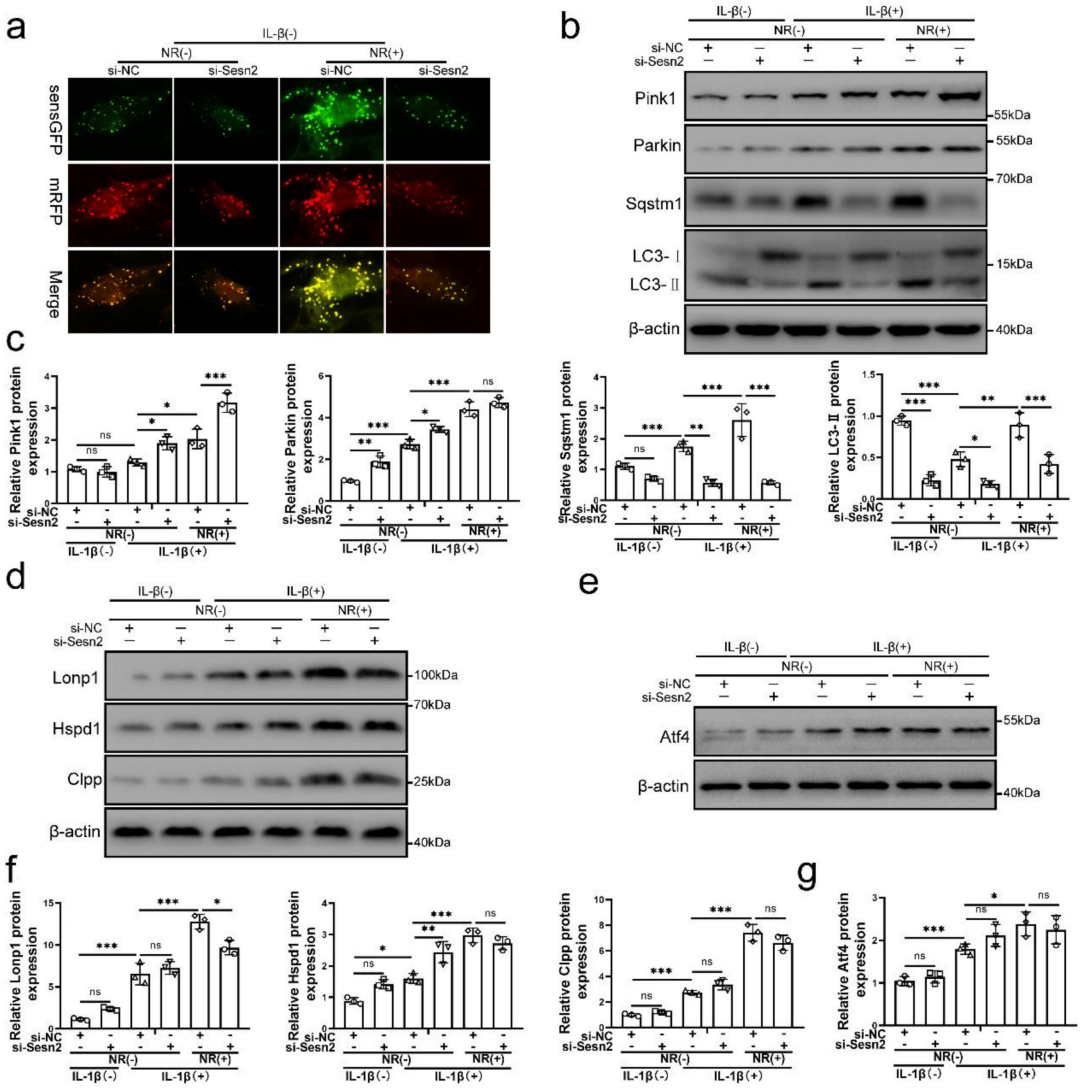
** Silencing of Sesn2 inhibited mitophagy induced by UPR^mt^ in NP cells. (a)** NP cells stably expressing the stubRFP-sensGFP-LC3 fusion protein were established and observed by the fluorescence microscope (n = 3). sensGFP is sensitive to the pH changes owing to the fusion of autophagosomes and lysosomes, whereas mRFP is stable. When autophagy was induced, autophagosomes and lysosomes were fused, sensGFP was quenched and mRFP was increased. The experimental grouping settings were indicated. (**b-g**) NP cells were transfected with si-NC or si-Sesn2, treated with 0 ng/ml and 20 ng/ml IL-1β for 48h and then treated with 0- and 1-mM NR for 6h. The experimental grouping settings were indicated. **(b)** Pink1, Parkin, Sqstm1, LC3 and β-actin were confirmed by Western blot (n = 3). **(c)** The quantitative analysis of Pink1, Parkin, Sqstm1 and LC3-Ⅱ protein expression were showed. **(d)** The protein expression of UPR^mt^ markers including Lonp1, Hspd1, Clpp and β-actin were confirmed by Western blot (n = 3). **(e)** Atf4 and β-actin were confirmed by Western blot (n = 3). **(f)** The quantitative analysis of Lonp1, Hspd1 and Clpp protein expression were showed. **(g)** The quantitative analysis of Atf4 protein expression were showed. Statistical significance was analyzed by one-way ANOVA followed by a post hoc Tukey's test. All data were presented as mean ± SEM (error bars). *p < 0.05; **p < 0.01; ***p<0.001.

Sesn2 knockdown was able to inhibit the protective effect of UPR^mt^ on NP cells. Importantly, NR-induced mitophagy was restraint by si-Sesn2 (Fig. 4a, b and c). However, the reduction in LC3-Ⅱ and Sqstm1 caused by si-Sesn2 was not accompanied by the low expression of Pink1 and Parkin. Silencing of Sesn2 caused an increased expression of Pink1 and Parkin, which intrigued us (Fig. 4b and c). When Sesn2 was silenced, the protein level of UPR^mt^ such as Lonp1, Hspd1 and Clpp was not changed in NP cells (Fig. 4d and f). Silencing of Sesn2 did not change the protein expression of Atf4 in NP cells (Fig. 4e and g). These results suggested that UPR^mt^ induced mitophagy to protect NP cells via Sesn2. According to our hypothesis, UPR^mt^ might independently regulate Sesn2 and UPR^mt^ marker molecules through Atf4. Sesn2 did not regulate UPR^mt^ marker molecules. Sesn2-regulated mitophagy was one of the many downstream of UPR^mt^. Although the mechanism is not yet clear, UPR^mt^ could target mitophagy via Sesn2 pathway.

### UPR^mt^ upregulated Sesn2-mediated mitophagy through Eif2ak3/Eif2ak4-eIF2α-Atf4 signal pathway

**Figure 5:**
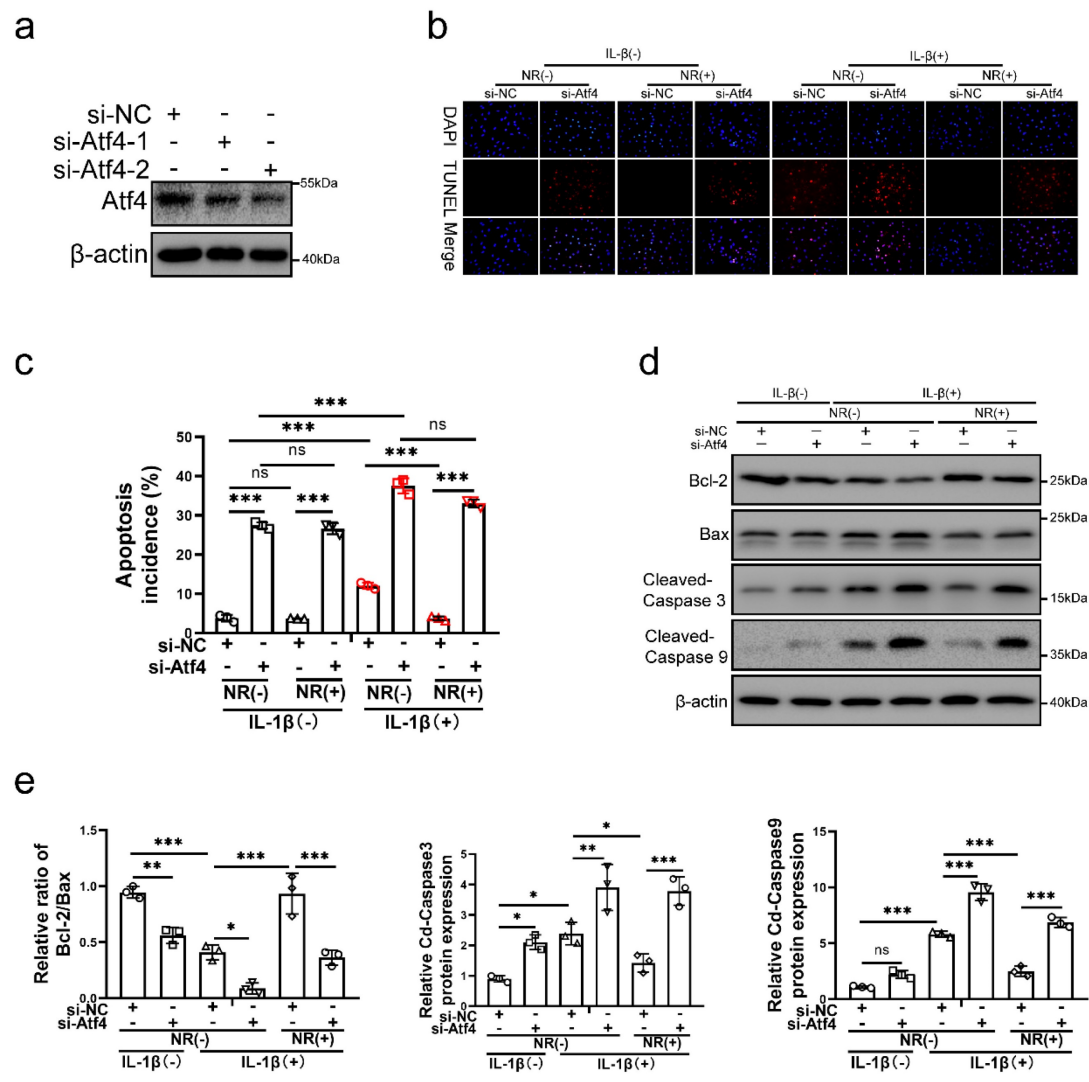
** Atf4 knockdown decreased the protective effect of NR in NP cells**. **(a)** Western blot of Atf4 and β-actin in NP cells (n = 3). NP cells were transfected with si-NC, si-Atf4-1 and si-Atf4-2 respectively. (b-f) NP cells were transfected with si-NC or si-Atf4, treated with 0 ng/ml and 20 ng/ml IL-1β for 48h and then treated with 0- and 1-mM NR for 6h. The experimental grouping settings were indicated.** (b)** TUNEL assays to NP cell apoptosis (n = 3). **(c)** The bar-chart of flow cytometry to NP cell apoptosis (n = 3). **(d)** Western blot to Bcl-2, Bax, Cleaved-caspase 3, Cleaved-caspase 9 and β-actin in NP cells (n = 3). **(e)** The quantitative analysis of Bcl-2/Bax, Cleaved-Caspase 3 and Cleaved-Caspase 9 protein expression were showed. Statistical significance was analyzed by one-way ANOVA followed by a post hoc Tukey's test. All data were presented as mean ± SEM (error bars). *p < 0.05; **p < 0.01; ***p<0.001.

To verify the role of Atf4 in UPR^mt^, Atf4 was silenced in NP cells before IL-1β and NR treatments. Atf4 was silenced by si-Atf4-1 and si-Atf4-2 in NP cells (Fig. 5a). NR could not protect NP cells from IL-1β after Atf4 knockdown (Fig. 5b and c). Reduction of apoptosis by NR were destroyed completely in NP cells after Atf4 knockdown. Regardless of whether it was treated by IL-1β, knocking down the expression of Atf4 also significantly increased the TUNEL positive rate of NP cells (Fig. 5b). Atf4 knockdown increased the apoptosis rate (late + early) from 3.83 ± 0.51% to 27.46 ± 1.19% in the IL-1β (-) + NR (-) groups. IL-1β increased the rate of apoptosis (late + early) from 3.83 ± 0.51% to 12.11 ± 2.90%. NR reduced apoptosis (late + early) induced by IL-1β from 12.11 ± 2.90% to 3.74 ± 0.38%. However, Atf4 knockdown increased the apoptosis rate (late + early) from 3.74 ± 0.38% to 33.09 ± 1.75% (Fig. 5c and Fig. S3a). The protein expression of Bcl-2, Bax and Cleaved-caspase3, Cleaved-caspase 9 bore out these results (Fig. 5d and e). These results revealed that Atf4 also reduced the protective effect of UPR^mt^ on NP cells. Knockdown of Atf4 damaged NP cells more severely than Sesn2, which meant in addition to regulating Sesn2, Atf4 might protect NP cells through UPR^mt^ markers or other ways. NR could protect partially NP cells after Sesn2 knockdown while NR could not protect completely NP cells after Atf4 knockdown.

### Atf4 was the common upstream of mitochondrial unfolded protein response marker molecules and Sesn2

**Figure 6:**
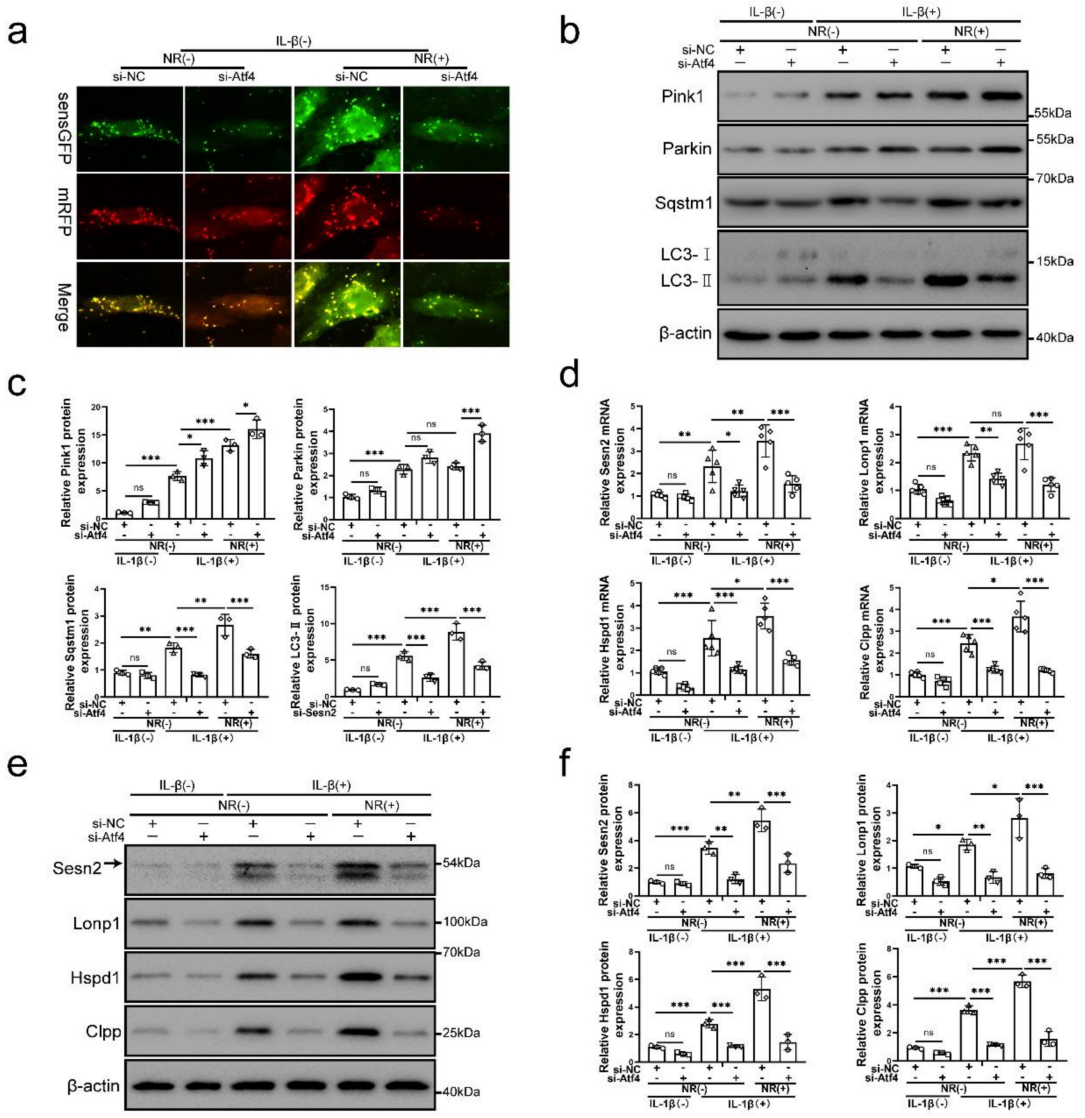
** Atf4 served as an upstream of Sesn2, mitophagy and UPR^mt^ markers. (a)** NP cells stably expressing the stubRFP-sensGFP-LC3 fusion protein were established and observed by the fluorescence microscope (n = 3). (b-g) NP cells were transfected with si-NC or si-Atf4, treated with 0 ng/ml and 20 ng/ml IL-1β for 48h and then treated with 0- and 1-mM NR for 6h. The experimental grouping settings were indicated. **(b)** Pink1, Parkin, Sqstm1, LC3 and β-actin were confirmed by Western blot (n = 3). **(c)** The quantitative analysis of Pink1, Parkin, Sqstm1 and LC3-Ⅱ protein expression were showed. **(d)** The mRNA expression of Sesn2, UPR^mt^ markers including Lonp1, Hspd1, Clpp were detected by qRT-PCR. **(e)** The protein expression of Sesn2, UPR^mt^ markers including Lonp1, Hspd1, Clpp and β-actin were confirmed by Western blot (n = 3). **(f)** The quantitative analysis of Sesn2, Lonp1, Hspd1 and Clpp protein expression were showed. Statistical significance was analyzed by one-way ANOVA followed by a post hoc Tukey's test. All data were presented as mean ± SEM (error bars). *p < 0.05; **p < 0.01; ***p<0.001.

To further investigate the underlying mechanism of Atf4 in this process, the level of mitophagy, Sesn2 and UPR^mt^ markers were confirmed in NP cells after Atf4 knockdown. Atf4 knockdown inhibited mitophagy induced by IL-1β and NR (Fig. 6a, b and c). Atf4 knockdown reduced Sqstm1 and LC3-Ⅱ in NP cells after IL-1β and NR treatments. However, Pink1 and Parkin expression was increased in NP cells after Atf4 knockdown (Fig. 6b and c). This phenomenon was consistent with the findings that increased expression of Pink1 and Parkin were observed after Sesn2 knockdown (Fig. 5b and c). On the contrary, silencing of Atf4 could reduce the mRNA level of UPR^mt^ markers and Sesn2 (Fig. 6d). Silencing of Atf4 also caused a reduction of UPR^mt^ markers and Sesn2 protein expression (Fig. 6e and f). In summary, these results demonstrated that UPR^mt^ upregulated Sesn2 expression through Eif2ak3/Eif2ak4-eIF2α-Atf4 signal pathway. Atf4 was the common upstream of UPR^mt^ marker molecules and Sesn2. Atf4 not only protect NP cells through Sesn2-induced autophagy, it might be protected by UPR^mt^ markers.

### NR cannot attenuate the IVDD of Sesn2 KO mice through mitophagy

**Figure 7:**
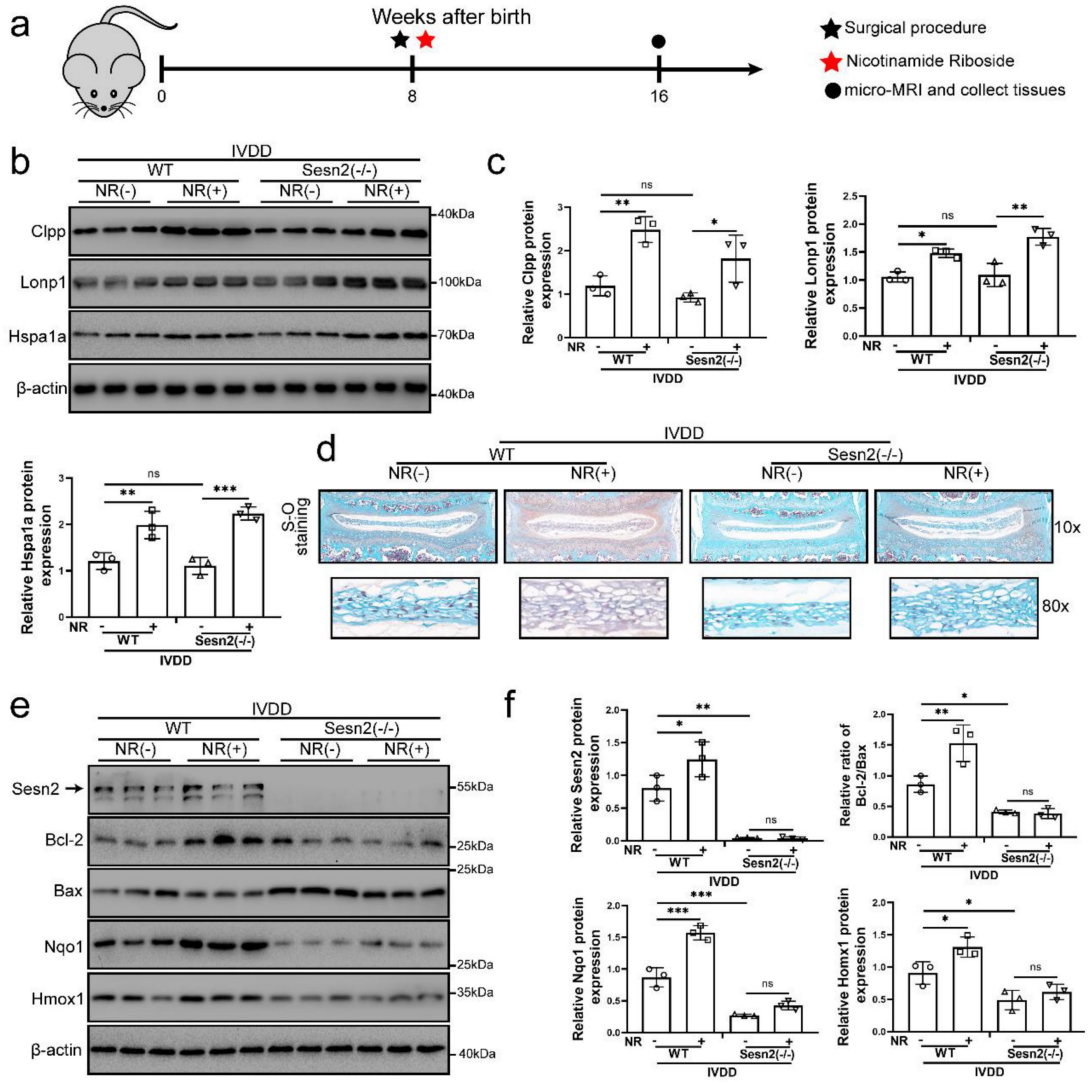
** NR could not attenuate IVDD of Sesn2 KO mice. (a)** Schematic diagram of animal experimental schedule. **(b)** UPR^mt^ markers including Clpp, Lonp1, Hspa1a and β-actin protein expression were confirmed by Western blot. **(c)** The quantitative analysis of Clpp, Lonp1 and Hspa1a protein expression were showed. **(d)** Safranin O-fast green staining showed the structure of the intervertebral disc of mice (n = 6). **(e)** Western blot to Sesn2, Bcl-2, Bax, Nqo1, Homx1 and β-actin in the tissue samples of mouse discs (n = 3). **(f)** The quantitative analysis of Sesn2, Bcl-2/Bax, Nqo1 and Homx1 protein expression were showed. Statistical significance was analyzed by one-way ANOVA followed by a post hoc Tukey's test. All data were presented as mean ± SEM (error bars). *p < 0.05; **p < 0.01; ***p<0.001.

For determining the role of Sesn2 between UPR^mt^ and mitophagy* in vivo*, we built Sesn2-/- mice and established IVDD model of Sesn2-/- mice (Fig. 7a). Sesn2 was not expressed in Sesn2-/- mice (Fig. S4a and b). Sesn2 knockout could not decrease UPR^mt^ genes Clpp, Hspa1a and Lonp1 expression (Fig. 7b and c). Interestingly, Sesn2-/- mice with IVDD and treated with NR demonstrated an increased expression of UPR^mt^ markers (Fig. 7b and c). These results proved *in vivo* that Sesn2 could not regulate UPR^mt^ markers.

Normal nucleus pulposus of intervertebral disc is rich in proteoglycan. As a result, normal nucleus pulposus is stained red by safranin O. When the nucleus pulposus degenerates or is injured, proteoglycans in the nucleus pulposus are reduced, resulting in light or no staining of safranin O. In figure 7d, the proteoglycan in the nucleus pulposus of intervertebral disc was significantly decreased and the height of the nucleus pulposus was significantly reduced after surgery procedure. Intervertebral discs form of lumbar in WT mice fed with NR exhibited a more well-developed NP and a higher disc height than WT mice without NR treatments after surgery procedure (Fig. 7d). NR also promoted the levels of proteoglycan. Intervertebral discs from Sesn2-/- mice after surgery procedure suffered worse histological disrupted. Knockout of Sesn2 further reduced the level of proteoglycan and the height of the nucleus pulposus. Importantly, NR was able to attenuate the degeneration of intervertebral discs in WT mice but not Sesn2-/- mice (Fig. 7d). Meanwhile, NR could not protect intervertebral disc cell from apoptosis and could not enhance antioxidant genes expression too (Fig. 7e and f). There was a remarkable reduction in Nqo-1 and Homx-1 as well as increase in apoptotic markers in Sesn2-/- mice and Sesn2-/- + NR. Furthermore, NR could not promote mitophagy in the intervertebral disc of Sesn2-/- mice (Fig. S4c and d). These results confirmed that Sesn2 could not regulate UPR^mt^ in the intervertebral disc but served as a downstream of UPR^mt^. Sesn2 was a bridge between UPR^mt^ and mitophagy.

Sqstm1 and LC3-Ⅱ expression were decreased in Sesn2-/- mice while Parkin and Pink1 were increased in Sesn2-/- mice (Fig. S4e and f). To further confirm the function of Sesn2 in the association of UPR^mt^ and mitophagy, Sesn2 was replied to express in the intervertebral disc of Sesn2-/- mice via injection of Lent-Sesn2. Recovery of mitophagy and reduction of apoptosis were observed in the intervertebral discs of Sesn2-/- mice after Sesn2 overexpression (Fig. S4e and f). Apoptotic genes Cleaved-caspase 3 and Cleaved-caspase 9 was increased in Sesn2-/- mice (Fig. S4e and f). Sesn2 overexpression increased Parkin, Sqstm1 and LC3-Ⅱ expression in Sesn2-/- (Fig. S4e and f). These results showed that mitophagy was decreased in the intervertebral disc of Sesn2-/- mice.

### Sesn2 interacted with Sqstm1 and Parkin respectively in NP cells

**Figure 8:**
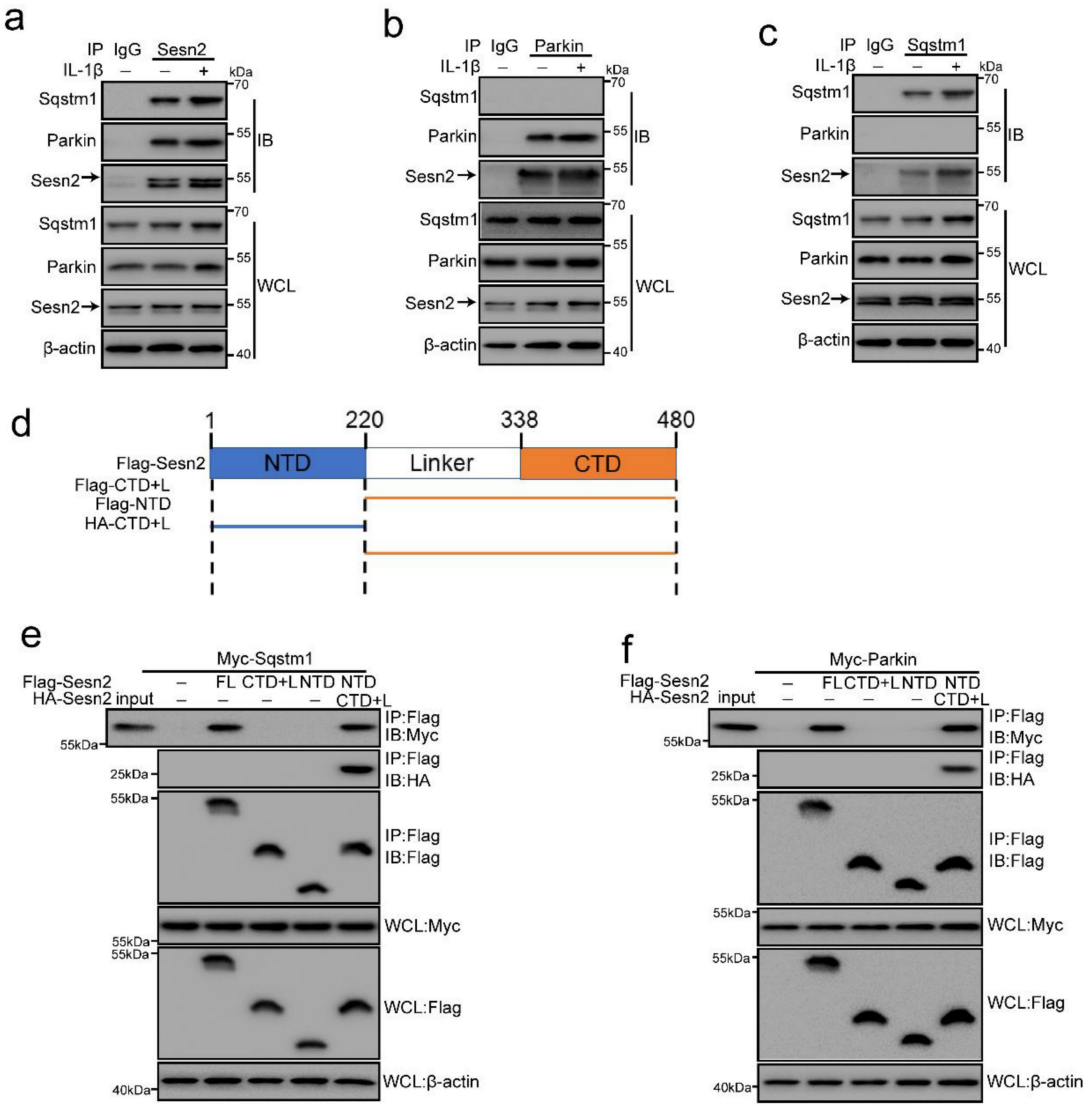
** Sesn2 served as a Parkin and Sqstm1 interacting protein. (a)** The interaction between Sesn2 and Parkin, Sqstm1 in NP cells treated with 0 or 20 ng/ml IL-1β for 48h was determined by Co-IP assays. Whole cell lysate (WCL) was used to perform IP of control serum (IgG) or anti-Sesn2 antibody, and then immunoblotting (IB) with indicator antibody to confirm the presence of a specific protein in the lysate. **(b)** The interaction between Parkin and Sesn2, Sqstm1 in NP cells treated with 0 or 20 ng/ml IL-1β for 48h was determined by Co-IP assays. WCL was used to perform IP of control serum (IgG) or anti-Parkin antibody, and then immunoblotting (IB) with indicator antibody to confirm the presence of a specific protein in the lysate. **(c)** The interaction between Sqstm1 and Sesn2, Parkin in NP cells treated with 0 or 20 ng/ml IL-1β for 48h was determined by Co-IP assays. WCL was used to perform IP of control serum (IgG) or anti-Sqstm1 antibody, and then immunoblotting (IB) with indicator antibody to confirm the presence of a specific protein in the lysate. **(d)** Sesn2 is a 55kda monomer, full alpha-helix, globular protein, containing different N-terminal [NTD, residues 66-220] and C-terminal [CTD, residues 339-480] domains connected by a partially disordered, partially helical linker region [linker, remaining 221-338]. **(e)** Immunoprecipitation of Sesn2 N- and C- terminal fragments. Transient transfection of full-length Flag-Sesn2 (FL), Flag-Sesn2-NTD (N-terminal domain,1-220), Flag-Sesn2-CTD + L (C-terminal domain plus Linker, 220-480) or Flag-Sesn2-NTD+HA-Sesn2-CTD +L and Myc-Sqstm1 to NP cells were treated with 20 ng/ mL IL-1β to prepare immunoprecipitation cell lysate. Immunoprecipitation and WCL were analyzed by Western blot for indicator protein detection results as shown (n = 3). **(f)** Immunoprecipitation of Sesn2 N- and C- terminal fragments. Transient transfection of full-length Flag-Sesn2 (FL), Flag-Sesn2-NTD (N-terminal domain,1-220), flag-Sesn2-CTD + L (C-terminal domain plus Linker, 220-480) or Flag-Sesn2-NTD+HA-Sesn2-CTD +L and Myc-Parkin to NP cells were treated with 20 ng/ mL IL-1β to prepare immunoprecipitation cell lysate. Immunoprecipitation and WCL were analyzed by western blotting for indicator protein detection results as shown (n = 3).

It is reported in the literature that Sesn2 can regulate autophagy and mitophagy through Sqstm1 and Parkin 41, 42, 47. Sesn2 can degrade Keap1 through Sqstm1-dependent autophagy, thereby increasing the level of Nrf2 in the cell 47. Sesn2 can also promote the translocation of Parkin to mitochondria and induce mitophagy 41. However, the interaction mechanism between Sesn2, Sqstm1 and Parkin has not yet been elucidated. Herein, Sesn2 was found to pull down Sqstm1 and Parkin (Fig. 8a). IL-1β enhanced the interaction Sesn2 with Sqstm1 and Parkin. This indicates that there might be an interaction between the three. However, it was interesting that Sqstm1 could pull down Sesn2, but not Parkin (Fig. 8b). Parkin was also able to pull down Sesn2, but not Sqstm1 (Fig. 8c). It showed that Sesn2, Sqstm1 and Parkin were not interacted together, but Sesn2 combined with Sqstm1 and Parkin respectively.

Sesn2 is a 55kda monomer, full alpha-helix, globular protein, containing different N-terminal [NTD, residues 66-220] and C-terminal [CTD, residues 339-480] domains connected by a partially disordered, partially helical linker region [linker, remaining 221-338] (Fig. 8d) 54. In order to test the importance of intramolecular contact between the two domains of Sesn2, we expressed the two region of the labeled N- and C-terminus as separate polypeptides separately or together, and performed co-immunoprecipitation analysis. Although neither domain alone could bind Sqstm1 and Parkin, when the two separated domains are expressed together, they had strong binding to each other (Fig. 8e and f). Similarly, although the two regions of Sesn2 alone could not bind to Sqstm1 and Parkin, the two co-expressed regions could bind to Sqstm1 and Parkin (Fig. 8e and f). Therefore, the stable interaction between the N-terminal and C-terminal domains of Sesn2 was necessary for the interaction with Sqstm1 and Parkin.

### Sesn2 promoted the translocation of cytosolic Parkin and Sqstm1 to the defective mitochondria to induce mitophagy

**Figure 9:**
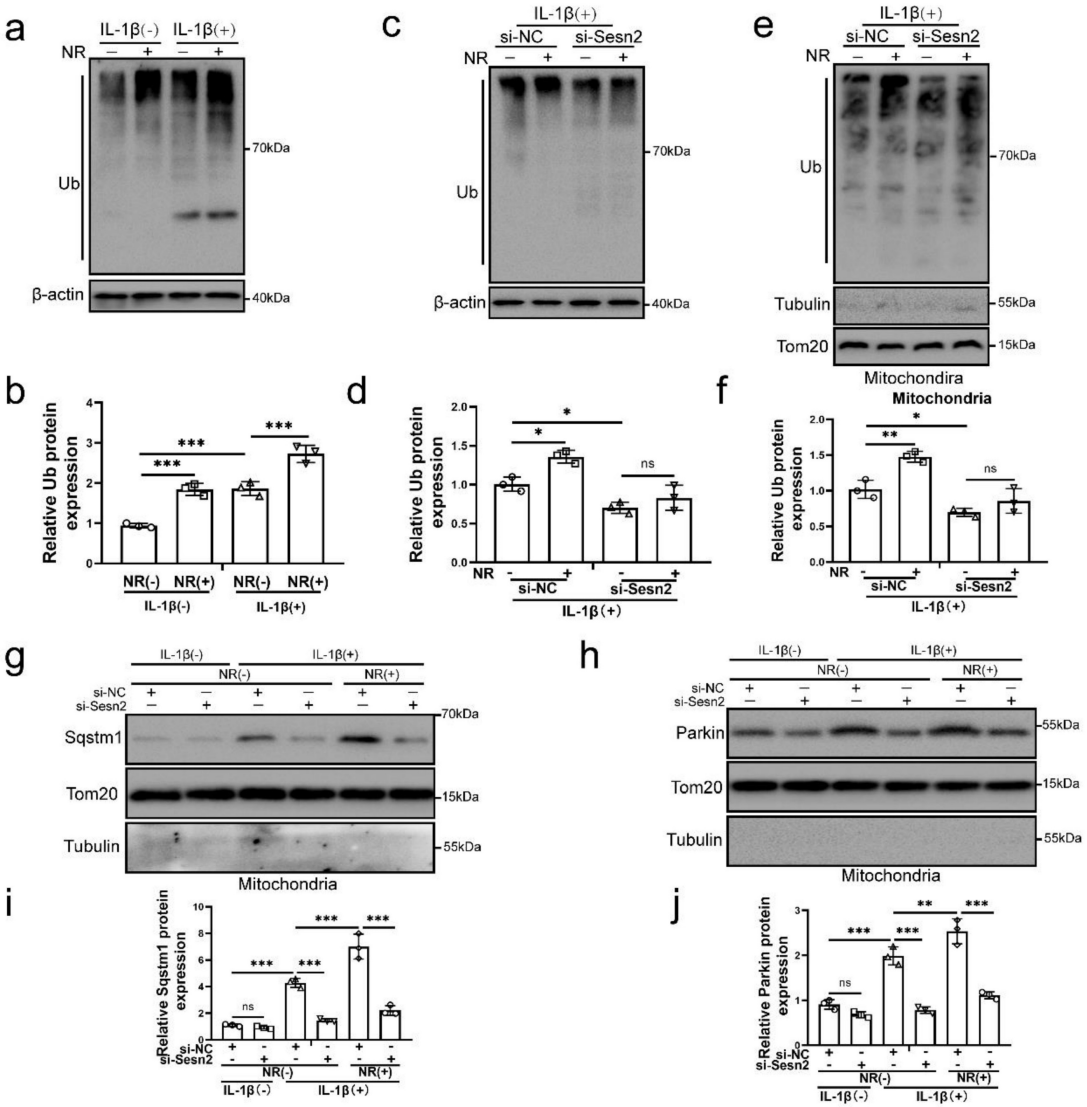
** Sesn2 improved the ubiquitination of mitochondria by promoting the transfer of Sqtms1 and Parkin to mitochondria. (a)** The level of ubiquitination in NP cells was determined by Western blot (n = 3). The NP cells were divided into 2 groups, which were treated with 0 ng/ mL and 20 ng/ mL IL-1β for 48h, respectively, and then each group was further divided into 2 groups, which were treated with 0- and 1-mm NR for 6h, respectively. **(b)** The quantitative analysis of Ub protein expression was showed. **(c)** The level of ubiquitination in NP cells was determined (n = 3). NP cells were divided into two groups, transfected with si-NC and si-Sesn2, and all groups were received 20 ng/ml IL-1β treatment for 48h. And then each group was divided into two groups, treated with 0- and 1-mM NR for 6h. **(d)** The quantitative analysis of Ub protein expression was showed. **(e)** The ubiquitination level of mitochondria in NP cells was detected (n = 3). NP cells were divided into two groups, transfected with si-NC and si-Sesn2, and all groups were received 20 ng/ml IL-1β treatment for 48h. And then each group was divided into two groups, treated with 0- and 1-mM NR for 6h. **(f)** The quantitative analysis of Ub protein expression was showed. (g-j) NP cells were transfected with si-NC or si-Sesn2, treated with 0 ng/ml and 20 ng/ml IL-1β for 48h and then treated with 0- and 1-mM NR for 6h. The experimental grouping settings were indicated. **(g)** The protein expression of Sqstm1 was determined by Western blot (n = 3). **(h)** The protein expression of Parkin was determined by Western blot (n = 3). **(i)** The quantitative analysis of Sqstm1 protein expression was showed. **(j)** The quantitative analysis of Parkin protein expression was showed. Statistical significance was analyzed by one-way ANOVA followed by a post hoc Tukey's test. All data were presented as mean ± SEM (error bars). *p < 0.05; **p < 0.01; ***p<0.001.

Mitochondrial ubiquitination plays an important role in mitophagy. Parkin is an E3 ubiquitin ligase. When mitochondrial fusion is blocked, mitochondrial subgroups are damaged and lose membrane potential, Parkin will translocation to damaged mitochondrial subgroups and mediate mitochondrial ubiquitination, thereby mediating mitophagy 55-57. The results of our study found that NR and IL-1β increased the ubiquitination level of NP cells (Fig. 9a and b). After Sesn2 was knockdown, the ubiquitinated level in NP cells was weakened (Fig. 9c and d). Importantly, knockdown of Sesn2 reduced the mitochondrial ubiquitination of NP cells induced by NR and IL-1β (Fig. 9e and f). In order to further determine the relationship between Sesn2, Sqstm1 and Parkin in mitophagy, mitochondrial proteins were extracted from NP cells. Silencing of Sesn2 reduced the level of Sqstm1 and Parkin on the mitochondrial (Fig. 9g, h, i and j). These results demonstrated that Sesn2 interacted the cytosolic Parkin and Sqstm1 and translocated them to the defective mitochondria to induce mitochondrial ubiquitination, thereby increasing mitophagy.

### The interaction of Sesn2 with Parkin and Sqstm1 was independent of each other

**Figure 10:**
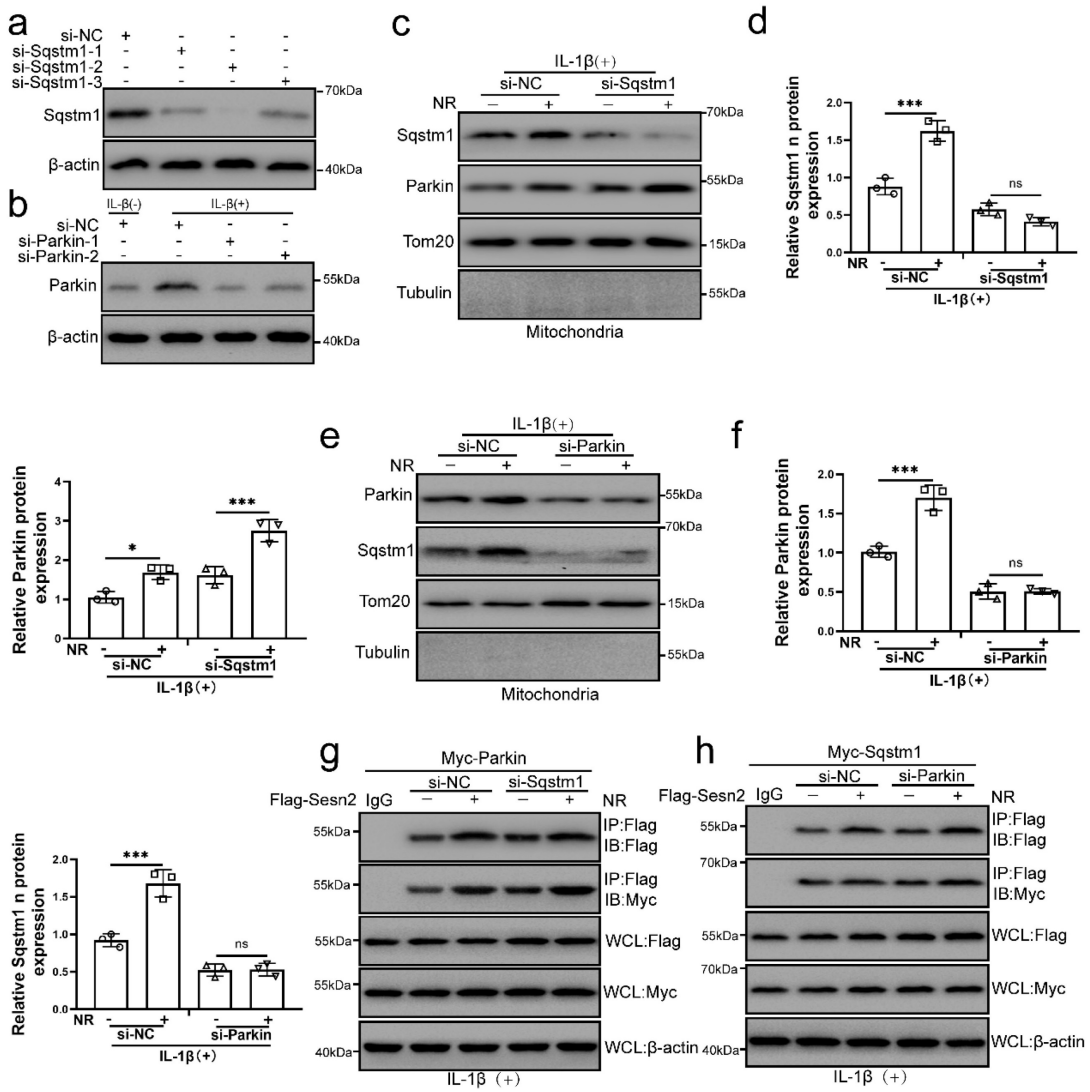
** The interaction of Sesn2 with Parkin and Sqstm1 was independent of each other. (a)** NP cells were transfected with si-NC, si-Sqstm-1, si-Sqstm-2 and si-Sqstm-3 respectively (n = 3). **(b)** NP cells were transfected with si-NC, si-Parkin-1 and si-Parkin-2 respectively (n = 3). NP cells were treated with 0 or 20 ng/ mL IL-1β for 48h and then 0- and 1-mm NR for 6h. The experimental grouping settings were indicated. **(c)** The protein level of Sqstm1 and Parkin in the mitochondria of NP cells were detected by Western blot (n = 3). NP cells were divided into two groups, transfected with si-NC and si-Sqstm1, and all groups were received 20 ng/ml IL-1β treatment for 48h. And each group was divided into two groups, treated with 0- and 1-mM NR for 6h. **(d)** The quantitative analysis of Sqstm1 and Parkin protein expression was showed. **(e)** The protein level of Sqstm1 and Parkin on the mitochondria of NP cells were detected (n = 3). NP cells were divided into two groups, transfected with si-NC and si-Parkin, and all groups were received 20 ng/ml IL-1β treatment for 48h. And then each group was divided into two groups, treated with 0- and 1-mM NR for 6h. **(f)** The quantitative analysis of Sqstm1 and Parkin protein expression was showed. **(g)** The level of Flag and Myc in WCL, IgG-IP and Flag-IP were determined respectively (n = 3). Firstly, Flag-Sesn2 and Myc-Parkin were transfected into NP cells and then transfected with si-NC and si-Pink1. All groups were received 20 ng/ml IL-1β treatment for 48h and then treated with 0 and 1mM NR for 6h. The experimental grouping settings were indicated. **(h)** The level of Flag and Myc in WCL, IG-IP and Flag-IP were determined respectively (n = 3). Firstly, Flag-Sesn2 and Myc-Sqstm1 were transfected into NP cells and then transfected with si-NC and si-Pink1. All groups were received 20 ng/ml IL-1β treatment for 48h and then treated with 0 and 1mM NR for 6h. The experimental grouping settings were indicated. Statistical significance was analyzed by one-way ANOVA followed by a post hoc Tukey's test. All data were presented as mean ± SEM (error bars). *p < 0.05; **p < 0.01; ***p<0.001.

The main role of Sqstm1 in mitophagy is to promote mitochondrial aggregation by positioning in the mitochondrial membrane, and further amplify the efficiency and effect of mitophagy 58, 59. Parkin is a key molecule that mediates mitochondrial autophagy 57. It was found that NR and IL-1β promoted the localization of Sqstm1 and Parkin on the mitochondria of NP cells. To investigate whether Sqstm1 and Parkin play a role in the interaction process of Sesn2, Sqstm1 and Parkin was silenced by si-Sqstm1 and si-Parkin respectively (Fig. 10a). We subsequently found that Sqstm1 knockdown would not affect the translocation of Parkin to mitochondria, while Parkin knockdown would affect the translocation of Sqstm1 to mitochondria (Fig. 10b, c, d and e). These results indicated that Parkin was an essential molecule for the translocation of Sqstm1 to mitochondria. Interestingly, Sqstm1 knockdown did not affect the interaction of Sesn2 and Parkin, meanwhile Parkin knockdown did not affect the interaction of Sesn2 and Sqstm1 too (Fig. 10f and g). The interaction of Sesn2 with Parkin and Sqstm1 was independent of each other.

### The interaction of Sesn2 with Parkin and Sqstm1 were independent of Pink1

**Figure 11:**
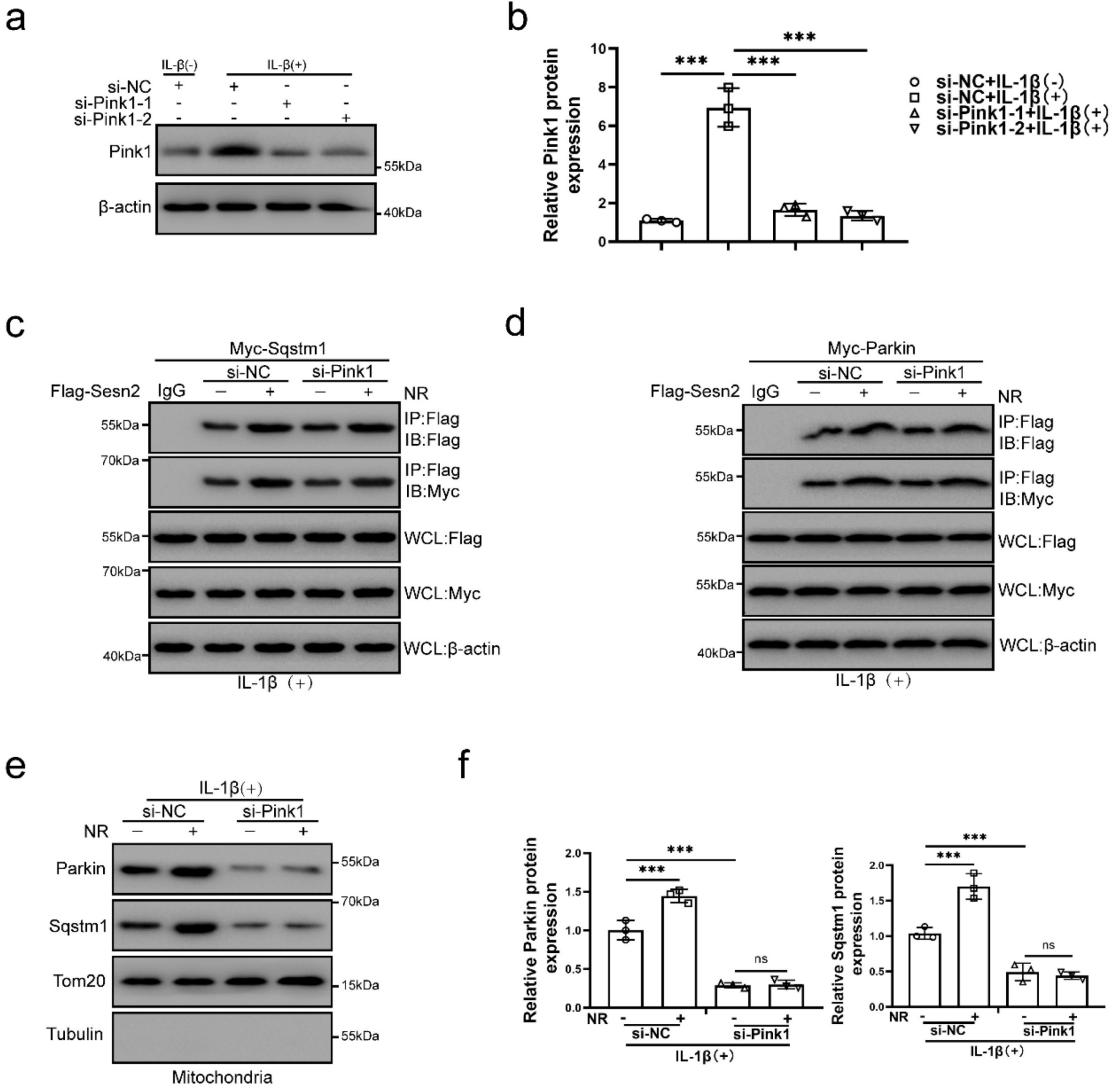
** The interaction of Sesn2 with Parkin and Sqstm1 were independent of Pink1**. **(a)** NP cells were transfected with si-NC, si-Pink1-1 and si-Pink1-2 respectively (n = 3). NP cells were treated with 0 or 20 ng/ml IL-1β treatment for 48h. The experimental grouping settings were indicated. **(b)** The quantitative analysis of Pink1 protein expression was showed. **(c)** The level of Flag and Myc in WCL, IG-IP and Flag-IP were detected respectively (n = 3). Firstly, Flag-Sesn2 and Myc-Sqstm1 were transfected into NP cells and then transfected with si-NC and si-Pink1. All groups were received 20 ng/ml IL-1β treatment for 48h and then treated with 0 and 1mM NR for 6h. The experimental grouping settings were indicated. **(d)** The level of Flag and Myc in WCL, IgG-IP and Flag-IP were detected respectively (n = 3). Firstly, Flag-Sesn2 and Myc-Parkin were transfected into NP cells and then transfected with si-NC and si-Pink1. All groups were received 20 ng/ml IL-1β treatment for 48h and then treated with 0 and 1mM NR for 6h. The experimental grouping settings were indicated. **(e)** The protein level of Sqstm1 and Parkin on the mitochondria of NP cells were detected (n = 3). NP cells were divided into two groups, transfected with si-NC and si-Pink1, and all groups were received 20 ng/ml IL-1β treatment for 48h. And then each group was divided into two groups, treated with 0- and 1-mM NR for 6h. **(f)** The quantitative analysis of Sqstm1 and Parkin protein expression was showed. Statistical significance was analyzed by one-way ANOVA followed by a post hoc Tukey's test. All data were presented as mean ± SEM (error bars). *p < 0.05; **p < 0.01; ***p<0.001.

Pink1 is a key molecule that induces Parkin to localize to damage mitochondria 60. To investigate the role of Pink1 in the interaction process, Pink1 was silenced by si-Pink1 in NP cells (Fig. 11a and b). However, we found that Pink1 knockdown did not affect the interaction of Sesn2 and Parkin (Fig. 11c and d). Silencing of Pink1 could reduce the location of Parkin and Sqstm1 on mitochondria (Fig. 11e and f). Based on the above results, we believed that Sesn2 promoted the translocation of Parkin and Sqstm1 to mitochondria, while Pink1 was the key molecule that attracted Parkin and Sqstm1 to transfer to mitochondria, but Pink1 did not affect the binding between Sesn2 with Parkin and Sqstm1. These results indicated that the interaction of Sesn2 with Parkin and Sqstm1 were independent of Pink1.

## Discussion

Both UPR^mt^ and mitophagy play an important role in mitochondrial quality control 6. During the mitochondrial dysfunction, UPR^mt^ has the protective effect on cellular and organismal physiology, as well as protects the mitochondria by mitochondrial stress responses such as mitophagy 61. UPR^mt^ and mitophagy, eliminating severe impaired mitochondrial, can be activated simultaneously because both have a response in similar forms of mitochondrial dysfunction 62-64. However, how they coordinate the recovery of the mitochondrial network remains to be determined.

The mechanism by which UPR^mt^ induces mitophagy is not yet clear. UPR^mt^ promotes the repair of mitochondrial function, improves the homeostasis of mitochondria, and removes unfolded, misfolded and ineffectively folded proteins within the mitochondria 65. What has caught our attention is that since the UPR^mt^ can protect the mitochondria, why does the UPR^mt^ inducer promote the activation of mitophagy? After mitochondrial dysfunction, UPR^mt^ induces molecular chaperone proteins, proteolytic enzymes, and mitophagy to repair mitochondrial functions and remove excessively damaged mitochondria 10. Multiple studies have shown that different forms of mitochondrial stress induce Atf4 and other protein molecules to participate in the expression of UPR^mt^ genes such as mitochondrial protein homeostasis and metabolic remodeling genes 66, 67. Atf4 and Eif2ak3 have been shown to mediate transcriptional induction of mitochondrial protein suppressor genes and the expression of genes involved in metabolic remodeling. However, it is not clear how transcription factor regulates mitochondrial dysfunction to achieve a specific mitochondrial stress response. Previous literatures have reported that Eif2ak3/Eif2ak4-eIF2α-Atf4 can induce the expression of Sesn2 18, 20, 21. The ectopic expression of Atf4 caused the transcriptional upregulation of Sesn2, while the ectopic expression of Atf3 and Chop did not cause the transcriptional upregulation of Sesn2, indicating that Atf4 regulates the expression of Sesn2^19^. The role of Atf4 in upregulation of Sesn2 gene induced by mitochondrial dysfunction were confirmed 20. Sesn2 is considered to be a key molecule that regulates mitophagy. Down-regulation of Sesn2 prevents Parkin from accumulating in damaged mitochondria 20. Sesn2 can be used as a positive regulator of Parkin-mediated mitophagy 42. In summary, we speculated that the UPR^mt^ mediated mitophagy through Eif2ak3/Eif2ak4-eIF2α-Atf4-Sesn2 signal pathway.

More and more studies have shown that IL-1β participates in the inflammatory response in the process of IVDD 68, 69. IL-1β is closely related to mechanical load, chronic inflammation, and participates in the vicious circle of IVDD 70. IL-1β, one of the commonly used inflammatory factors, is widely used to simulate NP cell *in vitro* 36, 71. Therefore, we used IL-1β to treat NP cells. To prove our hypothesis, we used the UPR^mt^ inducer NR to further activate the UPR^mt^ of NP cells. We found that IL-1β could induce the high expression of Sesn2, and NR could further promote the expression of Sesn2. At the same time, the expression trend of Eif2ak3/Eif2ak4-eIF2α-Atf4 signaling pathway is consistent with Sesn2. However, surprisingly, the expression of GCN2 and ATF4 in human IVDD tissues was inconsistent with the expression of SESN2. As the degree of IVDD increased, the expression of SESN2 gradually decreased while the expression of GCN and ATF4 gradually increased. It is reported in the literature that the eIF2α-Atf4 signaling pathway has the effect of protecting intervertebral disc cells in the early stage, and it can aggravate the damage of intervertebral disc cells in the late stage of degeneration 72. In order to further confirm our hypothesis, Sesn2 was silenced in NP cells and found that NR did not inhibit IL-1β-induced apoptosis after Sesn2 knockdown. But Sesn2 knockdown did not completely prevent the effect of NR. This suggested that UPR^mt^ does not completely protect NP cells through Sesn2. At the same time, the ability of NR to promote mitophagy was reduced. Silencing of Sesn2 did not change the level of UPR^mt^ and Atf4 protein expression. These results indicated that Sesn2 might be a bridge between UPR^mt^ and mitophagy. Knockdown of Atf4 has the same effect. However, Atf4 knockdown could completely destroy the effect of NR. Atf4 knockdown also reduced the protein level of UPR^mt^ marker such as Lonp1, Hspd1 and Clpp. Moreover, Atf4 knockdown also inhibit the protein expression of Sesn2 in NP cells. More importantly, in Sens2-/- mice, disc degeneration was more severe than in WT mice, and the expression level of mitophagy genes was reduced. After NR feeding, the degeneration of the disc in Sens2-/- mice was not alleviated. NR could promote the level of UPR^mt^ in the disc degeneration of Sesn2-/- mice, but could not increase the level of mitophagy in the disc degeneration of Sesn2-/- mice. It showed that UPR^mt^ induced mitophagy through Sesn2 to alleviate the degeneration of the intervertebral disc *in vivo*. It is reported in the literature that the Eif2ak3/Eif2ak4-eIF2α-Atf4 signaling pathway conducts the mediating signal pathway of the UPR^mt 6^. Mitochondrial dysfunction mediates UPR^mt^ through Eif2ak3/Eif2ak4-eIF2α-Atf4. The expression of UPR^mt^ genes restores mitochondrial function 6. This indicated that Sesn2 might be one of the signaling pathways mediated by the Eif2ak3/Eif2ak4-eIF2α-Atf4 signaling pathway. Atf4 was a key molecule of UPR^mt^ and Mitophagy coordinating effect, which communicates with mitophagy by Sesn2.

How does Sesn2 regulate mitophagy in NP cells? It is reported in the literature that Sesn2 can bind to Sqstm1-dependent autophagy to degrade Keap1, indicating that Sesn2 can bind to Sqstm1. Sesn2 can also accelerate the translocation of Parkin to mitochondria to promote mitophagy. We found that Sesn2 could interact with Sqstm1 and Parkin respectively. Although no single domain of Sesn2 could bind Parkin, when the two separated domains of Sesn2 were expressed together and they had strong binding to each other and to Parkin. Similarly, although the two separated domains of Sesn2 alone could not bind to Sqstm1, the two co-expressed domains were able to interact with Sqstm1. Therefore, the N-terminal and C-terminal domains of Sesn2 interacted stably, which were required for the interaction with Sqstm1 and Parkin. Sesn2 promoted the translocation of Sqstm1 and Parkin from the cytoplasm to the mitochondria. Parkin promoted the translocation of Sqstm1 to mitochondria, but Parkin did not affect the interaction of Sesn2 and Sqstm1. Pink1 also did not affect the interaction of Sesn2, Sqstm1 and Parkin in the cytoplasm. However, the translocation of Parkin and Sqstm1 to mitochondria needed to be mediated by Pink1.

**Figure 12:**
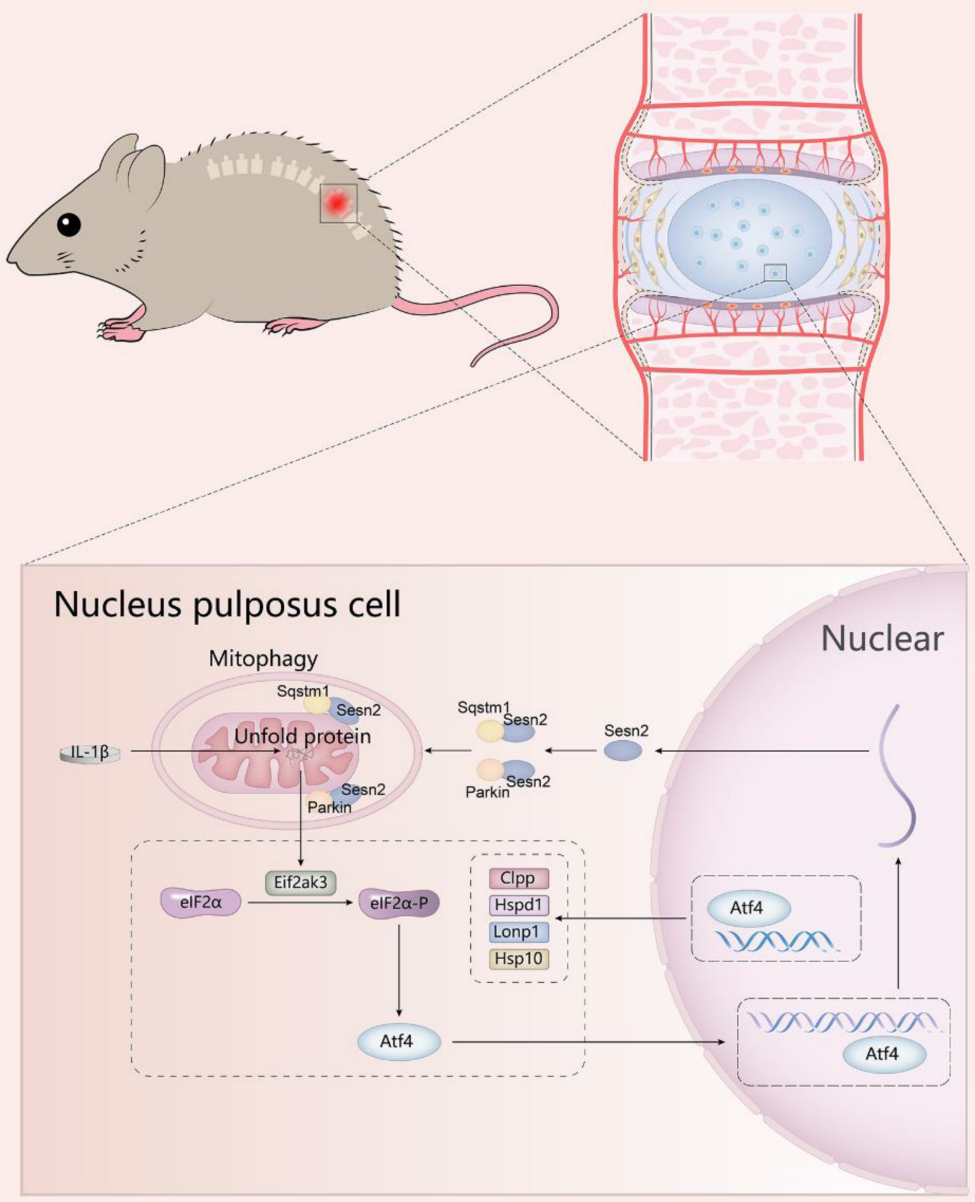
** A schematic diagram for the underlying mechanism of UPR^mt^ in regulating IVDD.** Mitochondrial unfold proteins stimulate UPR^mt^ marker genes and Sesn2 through Eif2ak3/Eif2ak4-eIF2α-Atf4 signal pathway. The whole process is called the mitochondrial unfolded protein response. Sesn2 acts as a bridge between UPR^mt^ and mitophagy. Sesn2 promoted the translocation of cytosolic Parkin and Sqstm1 to the defective mitochondria respectively, thereby enhancing mitophagy.

The translocation of Parkin to uncoupled mitochondria to induce mitophagy requires the activation of Pink1^73^. Pink1 is expressed and imported into all mitochondria, and then rapidly degraded by proteolysis and maintained at a very low level 74. When a group of mitochondria is damaged, Pink1 proteolysis is inhibited, and Pink1 is allowed to accumulate in the damaged mitochondria, and Parkin is then recruited to the mitochondria. Combined with our discovery, Pink1 was the key to regulating the translocation of Parkin to mitochondria. Pink1 accumulated on impaired mitochondria, attracting Parkin aggregation and Sesn2 could accelerate the aggregation of Parkin into the mitochondrial. Subsequently the aggregation of Parkin promoted Sqstm1 located on mitochondria and Sesn2 was also able to accelerate this process. The interaction of Sesn2, Parkin and Sqstm1 was independent of each other. Pink1 did not regulate the interaction of them (Fig. 12).

Why the protein expression of Pink1 and Parkin increased after Sesn2 knockdown? It is reported in the literature that Pink1 cannot successfully recruit Parkin into mitochondria, causing the expression of Pink1 and Parkin to gradually accumulate in the process of IVDD 75. Pink1 accumulates in continuously damaged mitochondria, while Parkin cannot accumulate in damaged mitochondria. Negative feedback regulation further promotes the expression of Parkin and Pink1. On the other hand, the further high expression mediates excessive mitophagy. Our previous research findings confirmed this phenomenon. In the late stage of IVDD, other factors induced excessive mitophagy may aggravate the IVDD 27. These two points explain why the expression of Pink1 and Parkin in human IVDD tissue samples increased with the increase of the degree of degeneration.

This study has the following points worthy of further improvement. Firstly, the role of UPR^mt^ maker genes including Clpp, Hspd1, Lonp1 and so on in the protection of IVDD need to be confirmed. Secondly, how Pink1 regulates the adsorption of Sqstm1 and Parkin on mitochondria needs to be further clarified in NP cells.

## Conclusion

UPR^mt^ induced mitophagy to reduce apoptosis and improve the metabolism of NP cells via Sesn2 signaling pathway, and then alleviates disc degeneration. Disturbances in the regulatory mechanisms of UPR^mt^ and mitophagy might be a risk factor in IVDD. UPR^mt^ agonist NR has the potential to become a drug for the treatment of IVDD.

## Supplementary Material

Supplementary figures.

## Figures and Tables

**Table 1:** The sequences of sgRNA

sgRNA	SgRNA sequence (5'-3')	PAM
SgRNA1	AAGAACTAAGAATGGTCGCT	GGG
SgRNA2	AGCTAGAGTACTTTGTTTGC	TGG
SgRNA3	AAGAATGGTCGCTGGGACTG	GGG
SgRNA4	AGTACTTTGTTTGCTGGAGT	AGG

**Table 2:** The sequences of siRNA

siRNA	Sense siRNA sequence (5'-3')	Antiense siRNA sequence (5'-3')
Si-Sesn2-1	GCGAGAUCAACAAAUUACU tt	AGUAAUUUGUUGAUCUCGC tt
Si-Sesn2-2	CCACGUUUGGAUAUGAAGA tt	UCUUCAUAUCCAAACGUGG tt
Si-Sesn2-3	GAAAGGAACCUCAAGAUCU tt	AGAUCUUGAGGUUCCUUUC tt
Si-Atf4-1	GCCUGACUCUGCUGCUUAU tt	AUAAGCAGCAGAGUCAGGC tt
Si-Atf4-2	GCCUAGGUCUCUUAGAUGA tt	UCAUCUAAGAGACCUAGGC tt
Si-Atf4-3	GAGUUAGUGUGACAGCUAA tt	UUAGCUGUCACACUAACUC tt
Si-Parkin-1	CCAAAUUGCUCAGGAAUAA tt	UUCUGA GUCACUCUUGCUG tt
Si-Parkin-2	CCAACUCCCUGAUUAAAGA tt	UCUUUAAUCAGGGAGUUGG tt

**Table 3:** The sequences of siRNA

siRNA	siRNA sequence (5'-3')
Si-Pink1-1	CGCAAATGTGCTTCATCTA
Si-Pink1-2	CCTATGAAATCTTCGGGCT
Si-Sqstm1-1	GCAUCUACAUUAAAGAGAA
Si-Sqstm1-2	GUGGGAACU CGCUAUAAGU
Si-Sqstm1-3	CCAACGUGAUUUGUGAUGG

**Table 4:** The sequence of primers

Gene	Forward (5'-3')	Reverse (5'-3')
Hspd1	CACCACCACTGCCACTGTTCTG	CAACAGCCAACATCACACCTCTCC
Clpp	ACTGTGCACACGGACAGTAG	CTGGATCTTTGGCCGGACTT
Lonp1	GGAGAAGACCATTGCGGCTA	AGATGTCGCGGTAGTGTTCC
Cox5a	TTGATGCCTGGGAGTTGCGTAAAG	ACAACCTCCAAGATGCGAACAGC
Sdhc	GCAAGAACCAAGACCGTCACTCC	TCCGACACTTGATGTGGGACCTAG
Ndufb5	CCGTTGCCTACCCTACTTCC	GGAGCGACAGTCTTCGGAAA
SESN2	CCGCTACATGACCTGACTCC	CTGCACATCACACACAAGCC
Sesn2	ACACCCCGACTACCTTAGCA	AACTCGGTCATGTGGGAACC
ATF4	TCAAACCTCATGGGTTCTCC	GTGTCATCCAACGTGGTCAG
GCN2	GCAGCAGCATGTGTGTGAAA	CTTGCAAAAGGGATCCGCAG
Keap1	AGCGCTACGATGTGGAAACA	GTCCAGGAACGTGTGACCAT
Nrf2	GACCTAAAGCACAGCCAACACAT	CTCAATCGGCTTGAATGTTTGTC

**Table 5:** Antibody information

Antibodies human (mouse or rat)	Source	Item No
Anti-SESN2 (Sesn2)	Proteintech	Cat No. 10795-1-AP
Anti-Bcl-2	Proteintech	Cat No. 26593-1-AP
Anti-Bax	Proteintech	Cat No. 50599-2-Ig
Anti-Sqstm1	Proteintech	Cat. No. 18420-1-AP
Anti-Tom20	Proteintech	Cat. No. 11802-1-AP
Anti-eIF2α	Cell Signaling Technology	Cat. No. #5324
Anti-p-eIF2α	Cell Signaling Technology	Cat. No. #3398
Anti-ATF4 (Atf4)	Cell Signaling Technology	Cat. No. #11815
Anti-GCN2 (Eif2ak3)	Cell Signaling Technology	Cat. No. #3302
Anti-p-JNK	Cell Signaling Technology	Cat. No. #4668
Anti-JNK	Cell Signaling Technology	Cat. No. #9252
Anti-Cleaved-Caspase 3	Cell Signaling Technology	Cat. No. #9661
Anti-Cleaved-Caspase 9	Cell Signaling Technology	Cat. No. #9507
Anti-Ubiquitin	Cell Signaling Technology	Cat. No. #3936
Anti-Parkin	Abcam	Cat. No. ab77924
Anti-Nqo1	Abcam	Cat. No. ab80588
Anti-Hmox1	Abcam	Cat. No. ab13248
Anti-Tubulin	Abcam	Cat. No. ab6160
Anti-LC3	Novus	Cat. No. NB100-2220
Mouse Anti-IgG	Cell Signaling Technology	Cat No. #5946
Mouse Anti-Sesn2	Proteintech	Cat No. 66297-1-Ig
Anti-β-actin	Beyotime Bitotechnology	Cat. No. AF0003
Mouse Anti-Flag	Cell Signaling Technology	Cat. No. #8146
Rabbit Anti-Flag	Cell Signaling Technology	Cat. No. #14793
Anti-Myc	abcam	Cat. No. ab32
Anti-HA	Sigma	Cat. No. H6533

## Abbreviations

UPR^mt^: Mitochondrial unfold protein response
IVDD: Intervertebral disc degeneration
NP: Nucleus pulposus
NR: Nicotinamide riboside
YME1L1: YME1-like 1 ATPase
Lonp1: Mitochondrial Lon protease-Like protein 1
CLPP: Caseinolytic mitochondrial matrix peptidase proteolytic subunit
HSPA9: Heat shock protein family A member 9
HSP70: Heat shock protein 70
ISR: Integrated stress response
eIF2α: Eukaryotic translation initiation factor 2 α subunit
PELD: Percutaneous Endoscopic Lumbar Discectomy
TLIF: Transforaminal Lumbar Interbody Fusion
HCG: human chorionic gonadotropin
CTD: C-terminal domain
NTD: N-terminal domain
S-O: safranin O-fast green
MRI: magnetic resonance imaging
