# Supplementary material for: Sesn2 Serves as a Regulator between Mitochondrial Unfolded Protein Response and Mitophagy in Intervertebral Disc Degeneration
Source: Int J Biol Sci. 2023 Jan 1;19(2):571–92. doi: 10.7150/ijbs.70211 (PMC9830501; doi:10.7150/ijbs.70211)
Supplement: Supplementary file 1 — Supplementary figures. [file ijbsv19p0571s1.pdf]

Supplementary Figure 1 (a) Flow cytometry to NP cell apoptosis (n = 3).

Supplementary Figure 2 (a) ROS assay kit was used to detect ROS production in NP cells. (b) JC-1 assay kit were used to detect mitochondrial membrane potential in NP cells. NP cells were transfected with si-NC or si-Sesn2, treated with 0 ng/ml and 20 ng/ml IL-1β for 48h and then treated with 0 and 1 mM NR for 6h. Statistical significance was analyzed by one-way ANOVA followed by a post hoc Tukey's test. All data were presented as mean ± SEM (error bars). *$p < 0.05$; **$p < 0.01$; ***$p<0.001$.

Supplementary Figure 3 (a) Flow cytometry to NP cell apoptosis (n = 3).

Supplementary Figure 4 (a) Western blot to Sesn2 and β-actin in WT, Sesn2+/- and Sesn2-/- mice (n = 3). (b) The quantitative analysis of Sesn2 protein expression was showed. (c) Mitophagy markers including Pink1, Parkin, Sqstm1, LC3 and β-actin (n = 3) protein expression were confirmed by Western blot. (d) The quantitative analysis of Pink1, Parkin, Sqstm1 and LC3-II protein expression was showed. (e) Western blot to Cleaved-caspase 3, Cleaved-caspase 9, Pink1, Parkin, Sqstm1 and β-actin. Littermates was divided into six WT and twelve Sesn2-/- mice as indicated. Twelve Sesn2-/- mice was divided randomly into six Sesn2-/- and four Sesn2-/- + Lenti-Sesn2 group as indicated. (f) The quantitative analysis of Cleaved-caspase 3, Cleaved-caspase 9, Pink1, Parkin and Sqstm1 protein expression was showed. Statistical significance was analyzed by one-way ANOVA followed by a post hoc Tukey's test. All data were presented as mean ± SEM (error bars). *$p < 0.05$; **$p < 0.01$; ***$p<0.001$.

Figure S1

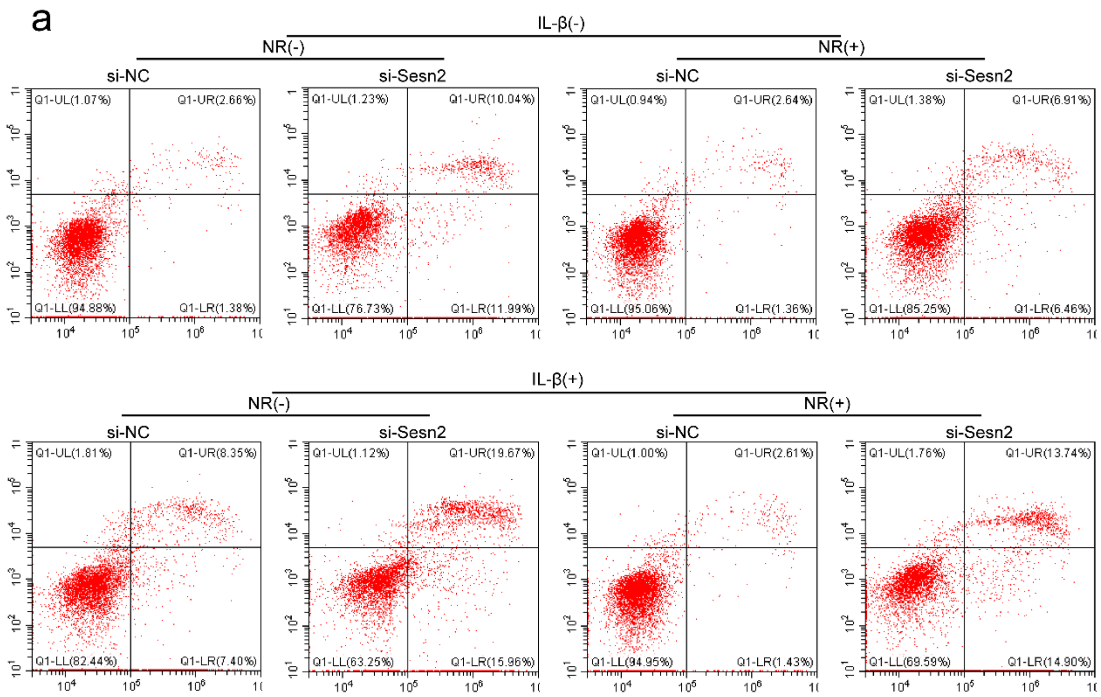

Figure S2

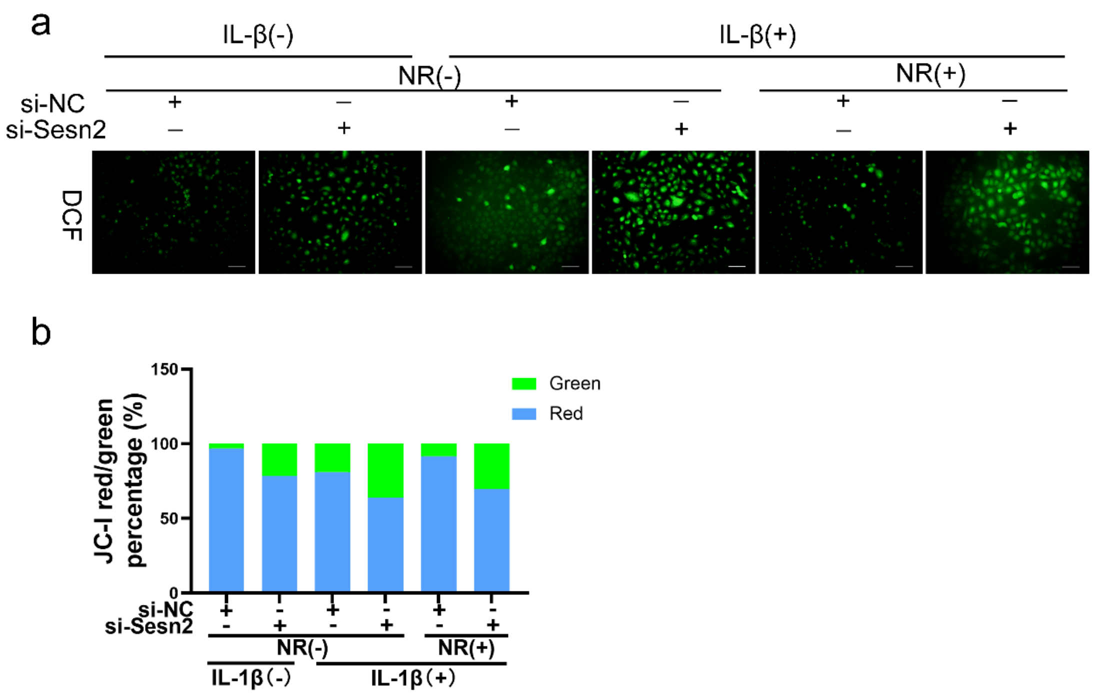

Figure S3

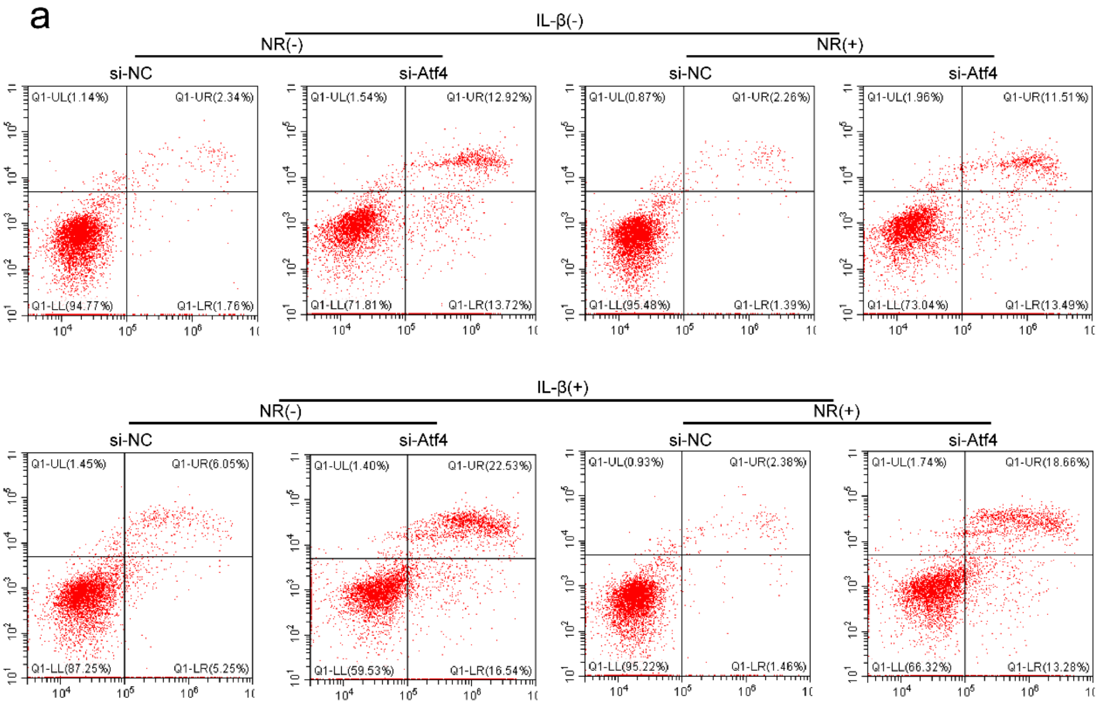
a
IL-β(-)
NR(-)
NR(+)
si-NC
si-Atf4
si-NC
si-Atf4
Q1-UL(1.14%) Q1-UR(2.34%) Q1-LL(94.77%) Q1-LR(1.76%)
Q1-UL(1.54%) Q1-UR(12.92%) Q1-LL(71.81%) Q1-LR(13.72%)
Q1-UL(0.87%) Q1-UR(2.26%) Q1-LL(95.48%) Q1-LR(1.39%)
Q1-UL(1.96%) Q1-UR(11.51%) Q1-LL(73.04%) Q1-LR(13.49%)
IL-β(+)
NR(-)
NR(+)
si-NC
si-Atf4
si-NC
si-Atf4
Q1-UL(1.45%) Q1-UR(6.05%) Q1-LL(87.25%) Q1-LR(5.25%)
Q1-UL(1.40%) Q1-UR(22.53%) Q1-LL(59.53%) Q1-LR(16.54%)
Q1-UL(0.93%) Q1-UR(2.38%) Q1-LL(95.22%) Q1-LR(1.46%)
Q1-UL(1.74%) Q1-UR(18.66%) Q1-LL(66.32%) Q1-LR(13.28%)

Figure S4

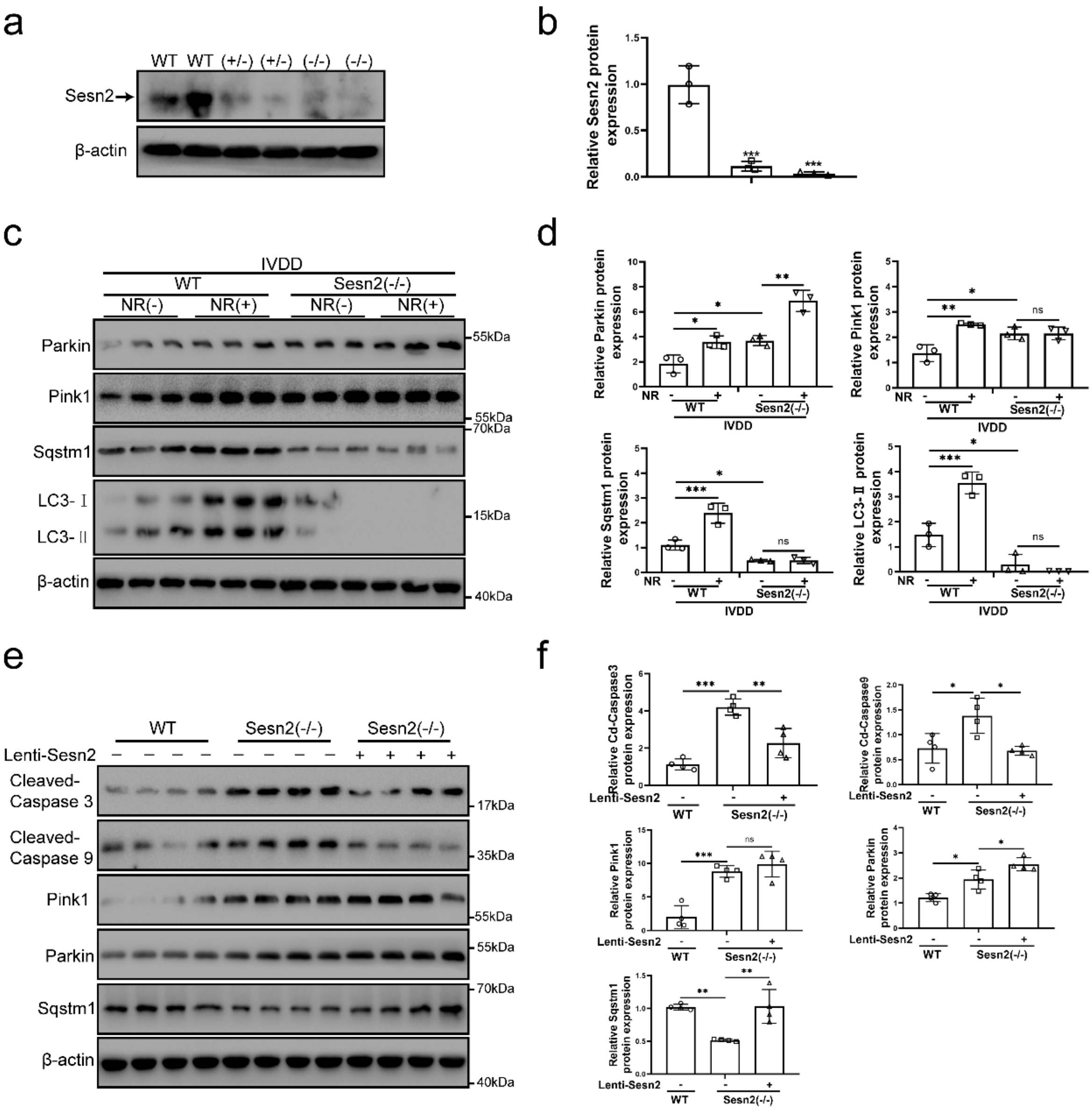
a
WT WT (+/-) (+/-) (-/-) (-/-)
Sesn2
β-actin
b
Relative Sesn2 protein expression
***
***
c
IVDD
WT
Sesn2(-/-)
NR(-) NR(+) NR(-) NR(+)
Parkin
Pink1
Sqstm1
LC3- I
LC3- II
β-actin
55kDa
55kDa
70kDa
15kDa
40kDa
d
Relative Parkin protein expression
Relative Pink1 protein expression
Relative Sqstm1 protein expression
Relative LC3-II protein expression
NR
WT
Sesn2(-/-)
IVDD
ns
e
WT
Sesn2(-/-)
Sesn2(-/-)
Lenti-Sesn2
Cleaved-Caspase 3
Cleaved-Caspase 9
Pink1
Parkin
Sqstm1
β-actin
17kDa
35kDa
55kDa
55kDa
70kDa
40kDa
f
Relative Cd-Caspase3 protein expression
Relative Cd-Caspase9 protein expression
Relative Pink1 protein expression
Relative Parkin protein expression
Relative Sqstm1 protein expression
Lenti-Sesn2
WT
Sesn2(-/-)
ns
